# Genome-wide identification and characterization of *TCP* genes involved in ovule development of *Phalaenopsis equestris*

**DOI:** 10.1093/jxb/erw273

**Published:** 2016-08-19

**Authors:** Yu-Fu Lin, You-Yi Chen, Yu-Yun Hsiao, Ching-Yu Shen, Jui-Ling Hsu, Chuan-Ming Yeh, Nobutaka Mitsuda, Masaru Ohme-Takagi, Zhong-Jian Liu, Wen-Chieh Tsai

**Affiliations:** ^1^Institute of Tropical Plant Sciences, National Cheng Kung University, Tainan, Taiwan; ^2^Department of Life Sciences, National Cheng Kung University, Tainan, Taiwan; ^3^Orchid Research and Development Center, National Cheng Kung University, Tainan, Taiwan; ^4^Shenzhen Key Laboratory for Orchid Conservation and Utilization, The National Orchid Conservation Center of China and The Orchid Conservation and Research Center of Shenzhen, Shenzhen, China; ^5^Division of Strategic Research and Development, Graduate School of Science and Engineering, Satitama University, Saitama, Japan; ^6^Research Institute of Bioproduction, National Institute of Advanced Industrial Science and Technology, Tsukuba, Japan; ^7^The Center for Biotechnology and BioMedicine, Graduate School at Shenzhen, Tsinghua University, Shenzhen, China; ^8^College of Forestry, South China Agricultural University, Guangzhou, China

**Keywords:** Cell proliferation, functional characterization, genome-wide identification, ovule development, *Phalaenopsis equestris*, TEOSINTE-BRANCHED/CYCLOIDEA/PCF (TCP) proteins.

## Abstract

TEOSINTE-BRANCHED/CYCLOIDEA/PCF (TCP) proteins are plant-specific transcription factors known to have a role in multiple aspects of plant growth and development at the cellular, organ and tissue levels. However, there has been no related study of TCPs in orchids. Here we identified 23 TCP genes from the genome sequence of *Phalaenopsis equestris*. Phylogenetic analysis distinguished two homology classes of PeTCP transcription factor families: classes I and II. Class II was further divided into two subclasses, CIN and CYC/TB1. Spatial and temporal expression analysis showed that *PePCF10* was predominantly expressed in ovules at early developmental stages and *PeCIN8* had high expression at late developmental stages in ovules, with overlapping expression at day 16 after pollination. Subcellular localization and protein–protein interaction analyses revealed that PePCF10 and PeCIN8 could form homodimers and localize in the nucleus. However, PePCF10 and PeCIN8 could not form heterodimers. In transgenic *Arabidopsis thaliana* plants (overexpression and SRDX, a super repression motif derived from the EAR-motif of the repression domain of tobacco ETHYLENE-RESPONSIVE ELEMENT-BINDING FACTOR 3 and SUPERMAN, dominantly repressed), the two genes helped regulate cell proliferation. Together, these results suggest that *PePCF10* and *PeCIN8* play important roles in orchid ovule development by modulating cell division.

## Highlight

Of twenty-three TCP genes identified from the genome of *Phalaenopsis* orchid, *PePCF10* and *PeCIN8* play important roles in orchid ovule development by modulating cell division.

## Introduction

Plant architecture and organ shape depends on complex coordination of cell proliferation and cell differentiation in response to genetic and environmental cues (Ingram and Waite, 2006; Kieffer *et al.*, 2011). The final shape of shoot lateral organs, leaves and flowers is determined by coordinated growth after the initiation of primordia from shoot meristems in seed plants. This coordination involves the complex behavior of many transcription factors (TFs).

The *TCP* gene family was first described in 1999 and named after the first three characterized members, TEOSINTE BRANCHED1 (TB1) in maize, CYCLOIDEA (CYC) in snapdragon, and Proliferating Cell Factor (PCF) in rice (Cubas *et al.*, 1999). They are ancient plant-specific TFs that originated near the base of streptophytes about 650–800 million years ago (Mondragon-Palomino and Trontin, 2011). From amino acid sequence similarity of the TCP domain, TCP proteins can be divided into two major classes: class I (also known as the PCF or TCP-P class) and class II (also known as the TCP-C class) (Martín-Trillo and Cubas, 2010; Manassero *et al.*, 2013). TCP class II is further subdivided into the CINCINNATA (CIN) and CYC/TB1 subclades. In addition, several class II TCP members share an arginine-rich R domain and an ECE motif (a glutamic acid–cysteine–glutamic acid stretch), both of unknown biological function (Martín-Trillo and Cubas, 2010).

The TCP family of plant-specific TFs controls multiple traits in diverse plant species by controlling cell proliferation and differentiation. This control includes regulation of lateral branching, leaf development and senescence, flower development, gametophyte development, embryo growth, seed germination, circadian rhythm, hormone pathways and mitochondrial biogenesis (Aguilar-Martinez *et al.*, 2007; Koyama *et al.*, 2007; Martín-Trillo and Cubas, 2010; Aguilar-Martinez and Sinha, 2013; Balsemão-Pires *et al.*, 2013; Manassero *et al.*, 2013). Class I genes are suggested to promote plant growth and proliferation. The Arabidopsis multiple mutants (*AtTCP8*, *15*, *21*, *22*, and *23*) have fewer but larger leaves (Aguilar-Martinez and Sinha, 2013). However, most class I single mutants analysed have mild or no phenotypic defects, probably due to genetic redundancy (Martín-Trillo and Cubas, 2010). Class II genes are involved in lateral organ development, including CIN in snapdragon and CYC/TB1 clade genes controlling axillary meristem development, such as TB1 in maize (Lopez *et al.*, 2015). In Arabidospsis, a group of *CIN*-type genes (*AtTCP3*, *4*, *5*, *10*, and *13*) functions redundantly to control shoot lateral organ morphology by negative regulation of boundary-specific genes (Koyama *et al.*, 2010).

Orchidaceae is one of the largest families among the angiosperms, consisting of about 850 genera with 25 000 species (Dressler, 1993). They are well known for their broad geographic distribution, tremendous diversity in plant forms and growth habits, extraordinary diversity in flower morphology within the framework of a relatively conserved structure, initiation of ovule development precisely regulated by pollination, and high value of hybrids as a floricultural commodity. Despite the economic and biological significance of orchids, and the importance of TCP TFs in controlling floral architecture, the relationship between the function of TCP genes, flower morphology and reproductive development remains poorly understood in this family.

Recently, the whole genome of the orchid *Phalaenopsis equestris* was sequenced (Cai *et al.*, 2015). The information provides a great opportunity to identify and characterize TCP TFs in orchid. In this study, we identified 23 non-redundant genes encoding TCP TFs in the genome of *P. equestris*. Systematic analysis including phylogenetic analysis and expression profiling was performed. Furthermore, a class I PCF-like gene, *PePCF10*, and a class II CIN-like gene, *PeCIN8*, were functionally characterized by cellular localization, protein–protein interaction and, in transgenic Arabidopsis, by gain-of-function and loss-of-function constructs. Our findings indicate that the two genes play important roles in orchid ovule development by modulating cell division.

## Materials and methods

### Accession numbers

The nucleotide sequences reported in this paper have been submitted to GenBank under the following accession numbers: *PePCF1*, KT258871; *PePCF2*, KT258872; *PePCF3*, KT258873; *PePCF4*, KT258874; *PePCF5*, KT258875; *PePCF6*, KT258876; *PePCF7*, KT258877; *PePCF8*, KT228878; *PePCF9*, KT258879; *PePCF10*, KT258880; *PePCF11*, KT258881; *PeCIN1*, KT258882; *PeCIN2*, KT258883; *PeCIN3*, KT258884; *PeCIN4*, KT258885; *PeCIN5*, KT258886; *PeCIN6*, KT258887; *PeCIN7*, KT258888; *PeCIN8*, KT258889; *PeCIN9*, KT258890; *PeCYC1*, KT258891; *PeCYC2*, KT258892; *PeCYC3*, KT258893.

### Plant material

*Phalaenopsis* plants were grown in a greenhouse at National Cheng Kung University (NCKU) under natural light (photosynthetic photon-flux density 90 μmol m^–2^ s^–1^) and controlled temperature (23–27 °C).

### RNA preparation

The flower of *Phalaenopsis equestris* has two main petals with three sepals behind them. The median petal is highly modified into an enlarged petal, called a lip or labellum. The male and female reproductive parts are fused in a structure, the gynostemium or column, located at the center of the flower (see Fig. 3A). Definition of the developing flower bud stage (stage 1: 0–1mm; stage 2: 1–2mm; stage 3: 2–5mm; stage 4: 5–10mm; Fig. 3B) is based on the description by Tsai *et al.* (2004). Morphological changes in the ovary and ovule development at various days after pollination (DAP) is based on the description by Chen *et al.* (2012). For RNA extraction, samples were collected, immersed in liquid nitrogen, and stored at –80 °C as described (Tsai *et al.*, 2004).

### Sequence retrieval for TCP proteins

The conserved TCP DNA-binding domain based on the hidden Markov model (HMM) (PF03634) was obtained from the Pfam protein family database (http://pfam.sanger.ac.uk). To identify the TCP TF coding genes of *P. equestris*, the HHM profile of the TCP domain was used as a query for an HMMER search (http://hmmer.janelia.org/) of the *P. equestris* genome sequence (Cai *et al.*, 2015) (*E*-value=0.00001). Furthermore, to verify the reliability of the results, all candidate TCP sequences were analysed to confirm the presence of the conserved TCP domain by using InterproScan (Quevillon *et al.*, 2005). The sequences of TCP family members in the genome of Arabidopsis and *Oryza sativa* were retrieved from the PlantTFDB TF database (http://planttfdb.cbi.pku.edu.cn/, v3.0).

### Sequence alignment and phylogenetic analysis

Multiple sequence alignment of the amino acid sequences of TCP proteins in *P. equestris*, Arabidopsis and rice genomes involved use of Clustal W (Thompson *et al.*, 1994) with default settings. Subsequently, MEGA 4.0 (Tamura *et al.*, 2007) was used to construct an unrooted phylogenetic tree based on alignments with the neighbor-joining method and the parameters JTTmodel, pairwise gap deletion and 1000 bootstraps.

### Real-time quantitative RT-PCR

RNA samples were treated with RNase-free DNase (New England Biolabs, Hitchin, UK) to remove remnant DNA, then underwent synthesis of first-strand cDNA by the use of the SuperScript III reverse transcriptase (Invitrogen, Carlsbad, CA, USA). Gene-specific primers for *PeTCP* genes were designed with the use of Primer Express (Applied Biosystems, Foster City, CA, USA) and are listed in Supplementary Table S1 at *JXB* online. *PeActin4* (5′-TTGTGAGCAACTGGGATG-3′ and 5′-GCCACGCGAAGTTCATTG-3′) and *18S rRNA* (5′-TTAGGCCACGGAAGTTTGAG-3′ and 5′-ACACTTCACCG GACCATTCAA-3′) (Lin *et al.*, 2014) were used as internal control. Each real-time RT-PCR contained 5ng of cDNA, 20mM primers and 12.5ml of SYBR GREEN PCR Master Mix (Applied Biosystems), and water was added to 25ml. Real-time PCR involved use of the ABI 7 500 Real-Time PCR Instrument (Applied Biosystems). For each real-time RT-PCR, each sample was analysed in triplicate. Data were analysed by the use of the Sequencing Detection System v1.2.3 (Applied Biosystems). The software MultiExperiment Viewer was used to construct heatmap representations for expression patterns.

### Whole-mount *in situ* hybridization

*Phalaenopsis equestris* inflorescences were fixed in 4% (v/v) paraformaldehyde and 0.5% (v/v) glutaraldehyde for 24h at 4 °C. They were dehydrated through a graded ethanol series. Digoxigenin-labeled sense and antisense RNA probes containing a partial C-terminal region and 3′-UTR were synthesized following the manufacturer’s instructions (Roche Applied Science). Hybridization and immunological detection of the signals with alkaline phosphatase were as described by Traas (2008).

### Subcellular localization

Template-specific primers were designed by addition of attB1 adapter primer (5′-GGGGACAAGTTTGTACAA AAA AGCAGGCTCCACC-3′) to the 5′ end of the first 18–25 nt of each open reading frame (ORF), and attB2 adapter primer (5′-GGGGACCA CTTTGTACAAGAAAGCTGGGTT-3′) to the 3′ end of the first 18–25 nt of each ORF, which generated the full-length attB1 and attB2 sites flanking the ORFs (S1). Gateway-compatible amplified ORFs were recombined into the pDONR 221 vector (Invitrogen) by BP cloning. BP reactions were used directly for bacterial transformation. Entry clones were used for transformation of *E. coli* DH5α cells, and bacteria were plated on LB medium containing kanamycin. These entry clones were set up in LR reactions for recombining target genes into the destination vector. The 35S promoter-driven green fluorescent protein (GFP)-fusion construct for protoplast transfection was created by use of the high-copy plasmid p2GWF7, with C-terminal fusion (Karimi *et al.*, 2007). Entry clones were used for generation of GFP-tagged clones. Then, the LR reactions were used for transformation of *E. coli* DH5α. Transformants were selected on plates containing ampicillin. The plasmids were transfected into *Phalaenopsis* protoplasts by PEG transformation. Signals were visualized by confocal laser microscopy (Carl Zeiss LSM780, Jena, Germany).

### Bimolecular fluorescence complementation (BiFC) assay

Gateway-compatible vectors [pSAT5(A)-DEST-cEYFP-N1 and pSAT5-DEST-cEYFP-C1(B) (Lin *et al*., 2014)] were used to generate expression vectors by a Gateway cloning strategy. The N-terminus of yellow florescent protein (YFP) (YN) was cloned upstream of *PePCF10* and *PeCIN8* in the pE-SPYNE vector, and the C-terminus of yellow florescent protein (YC), was fused upstream of *PePCF10* and *PeCIN8* in the pE-SPYCE vector. The signals were visualized by confocal laser microscopy (Carl Zeiss LSM780).

### Yeast two-hybrid analysis and Quantification of β-galactosidase activity

For the yeast two-hybrid, the full-length coding sequences of *PePCF10* and *PeCIN8* were cloned into either pGBKT7 bait vector (fused with Gal4 DNA-binding domain; BD) or pGADT7 prey vector (fused with GAL4 activation domain (AD), Clontech, http://clontech.com). The constructs were verified by sequencing and co-transformed into yeast strain Y187. To test for interactions, the bait and prey constructs were co-expressed in yeast. The yeast cells were plated on selection medium containing SD/–Trp–Leu and then incubated in a growth chamber at 30 °C for 2–5 d (Li *et al.*, 2011). All primers used for cloning are listed in Supplementary Table S1. *β*-Galactosidase activity from cotransformed yeast was measured using the *o*-nitrophenyl *β*-D-galactopyranoside assay, where *o*-nitrophenyl produced from cleavage of *o*-nitrophenyl *β*-D-galactopyranoside by β-galactosidase was detected by spectrophotometry (Li *et al.*, 2011, Li & Zachgo, 2013). For systematically analysing interaction behavior of PePCF10, *PePCF10* was cloned into pDEST_GBKT7 bait vector, which was retrieved from Arabidopsis Biological Resource Center (ABRC) from the Gateway entry clone. The Y2H Gold yeast strain (Takara Bio Inc.) was employed for the assay. After selecting the yeast harboring bait plasmid, prey plasmids constructed on pDEST_GAD424 vector (Mitsuda *et al.*, 2010) were additionally transformed and spotted onto positive control medium, which lacks leucine and tryptophan, and test medium, which lacks histidine, leucine, and tryptophan. Yeast growth was observed daily for several days.

### Construction of transformed fusions and Arabidopsis transformation

cDNA fragments containing the coding regions of *PePCF10* and *PeCIN8* were cloned into the pBI121 vector (primers are in Supplementary Table S1). The constructs were then introduced into *Agrobacterium tumefaciens* (strain GV3101). To construct the transgene for the chimeric repressor, the coding sequences of *PePCF10* and *PeCIN8*, except for the stop codon (primers are in Supplementary Table S1), were cloned into the *Sma*I site of p35SSRDXG in-frame to the region that encodes the SRDX repression domain (LDLDLELRLGFA) from *SUPERMAN* (Hiratsu *et al.*, 2002, 2003). p35SSRDXG contains a CaMV 35S promoter followed by an Ω translation enhancer sequence, the SRDX repression domain sequence, Nos terminator and the *att*L1 and *att*L2 Gateway recombination sites (Invitrogen) outside the regions of the 35S promoter and the Nos terminator in the pUC119 vector. The transgene cassette was transferred into the destination vector pBCKK by the Gateway LR clonase reaction (Invitrogen). Each gene was used to transform *Arabidopsis thaliana* Col-0 by the floral dip method, as described previously (Clough and Bent, 1998). To select transformed Arabidopsis, seeds (T0) were screened on media supplemented with 50 µg ml^–1^ kanamycin (Sigma-Aldrich). After a 2-week selection, the kanamycin-resistant seedlings (T1) were transferred to soil and grown under the conditions described previously. Kanamycin segregation in the T1 generation was analysed by use of chi-square test. The homozygous, kanamycin-resistant T2 generation was used to confirm the integration fragment by PCR for each construct. Transformed lines with segregation ratio 3:1 were collected for analysis.

### Scanning electron microscopy

Leaves and flowers were frozen by liquid nitrogen and observed by scanning electron microscopy (Hitachi TM3000, Tokyo, Japan). The data were analysed by using ImageJ (http://rsb.info.nih.gov/ij/).

## Results

### Identification and sequence analyses of TCP genes from *P. equestris*

To identify TCP TF coding genes, the HMM profile of the TCP domain (PF03634) was blast searched in the *P. equestris* genome (Cai *et al.*, 2015). Full-length sequences of 23 TCP family genes were identified from the genome. All candidate TCP genes were confirmed to encode the conserved TCP domain with the use of InterproScan (Quevillon *et al.*, 2005). The mean length of the 23 TCP TFs was 307 amino acids (range 220–425 amino acids). Other characteristics, including isoelectric point, molecular mass, and location at *Phalaenopsis* scaffolds are shown in Table 1.

**Table 1.** *TCP gene family in* Phalaenopsis equestris

Gene Name	Length (a.a.)	Molecular mass (Da)	pI	Scaffold location
*PePCF1*	253	26 846.3	5.71	Scaffold000123:1543921~1544682
*PePCF2*	289	30 740.3	7.96	Scaffold000865:254675~255534
*PePCF3*	333	34 736.9	9.51	Scaffold000927:176~1177
*PePCF4*	301	31 308.5	9.09	Scaffold000053:108817~109722
*PePCF5*	303	32 070.5	6.26	Scaffold001019:11294~12205
*PePCF6*	251	27 085.3	6.7	Scaffold000382:232040~232795
*PePCF7*	257	27 559.3	6.75	Scaffold000552:2647726~2648558
*PePCF8*	230	24 376.1	9.24	Scaffold000002:70407263~70407955
*PePCF9*	274	27 428.5	9.74	Scaffold000002:66457383~66457997
*PePCF10*	370	38 951.1	7.93	Scaffold000058: 217817~218940
*PePCF11*	274	29 655.9	8.75	Scaffold000349:250533~251358
*PeCIN1*	392	42 192	6.65	Scaffold000552:3228304~3229491
*PeCIN2*	372	40 543.5	9.06	Scaffold000272:914770~915888
*PeCIN3*	363	40 087.8	8.79	Scaffold000113:35591~36682
*PeCIN4*	277	30 765.9	7.79	Scaffold000404:25049~25882
*PeCIN5*	292	32 589.4	6.38	Scaffold000873:1549183~1550061
*PeCIN6*	425	44 992.4	9.89	Scaffold000428:5478841~5480211
*PeCIN7*	324	35 144.8	6.7	Scaffold000198:252605~253579
*PeCIN8*	220	24 505.2	6.21	Scaffold000180:1361454~1362116
*PeCIN9*	310	34 721.9	7.84	Scaffold000408:191611~192543
*PeCYC1*	288	32 774.3	8.94	Scaffold000878:1078313~1079179
*PeCYC2*	334	37 454.1	9.07	Scaffold000428:1243571~1244575
*PeCYC3*	326	36 663.6	9.06	Scaffold000428:1253199~1254179

### Sequence comparison and phylogenetic analysis

**Fig. 1.**
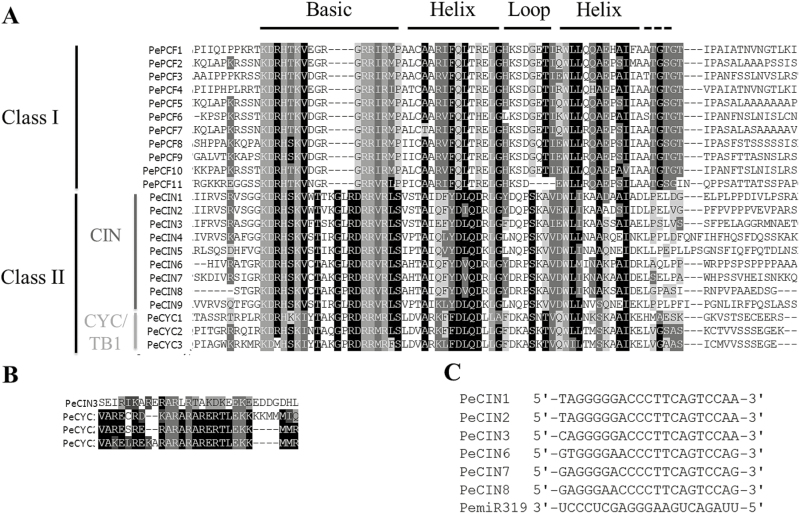
Multiple alignment of TCP protein sequences for *Phalaenopsis equestris*. (A) Alignment of the TCP domain and adjoining sequence for the predicted orchid TCP proteins. Overall conserved amino acids are in black. (B) Alignment of the R-domain of class II subfamily members. (C) Alignment of putative areas for *miR319*.

**Fig. 2.**
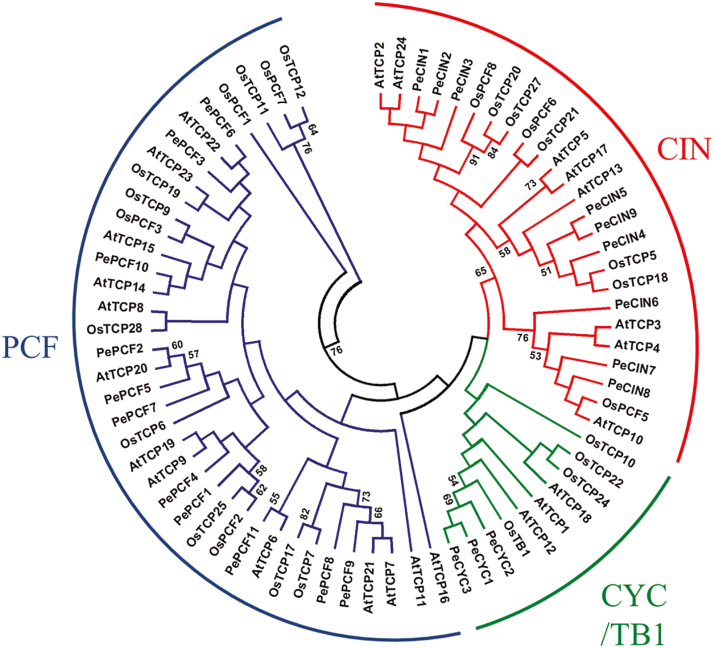
Phylogenetic relationships of TCP transcription factors from *P. equestris*, Arabidopsis and rice. The unrooted phylogenetic tree was created with MEGA 4.0 by the neighbor-joining method and the bootstrap test involved 1000 iterations. Blue, red and green lines indicate the PCF, CIN and CYC/TB1clades, respectively.

On multiple sequence alignment of the 23 PeTCP proteins, the sequences were found to encode a putative TCP-domain protein that contains a basic helix–loop–helix-type motif at the N-terminus (Supplementary Fig. S1). Orchid TCP proteins could be divided into two subfamilies, as for all species so far (Fig. 1A). The PCF or class I subfamily members showed an extended homology C-terminus from the TCP domain, and the class II subfamily had, as reported earlier, an extended four amino acids in the basic region. In addition, both subfamilies showed internally conserved but distinct loop region sequences (Cubas *et al.*, 1999). According to this division, 11 genes belonged to the class I subfamily and 12 to the class II subfamily in the *Phalaenopsis* orchid genome. The result is also supported by the phylogenetic analysis (Fig. 2). In addition, the class II subfamily could be further divided into two subclades, class IIa or CYC/TB1 and class IIb or CIN (Fig. 2). The CYC/TB1 subclade contains the three orchid genes *PeCYC1*, *PeCYC2*, and *PeCYC3* and CIN includes nine members, *PeCIN1*– *PeCIN9* (Figs 1A and 2).

Previous reports showed that Arabidopsis class II CYC/TB1 genes *AtTCP1*, *AtTCP12*, and *AtTCP18* as well as CIN genes *AtTCP2* and *AtTCP24* contain the R-domain at the C-terminus of the TCP domain (Yao *et al.*, 2007). In *Phalaenopsis*, all three genes *PeCYC1*, *PeCYC2*, and *PeCYC3* in CYC/TB1 and one gene, *PeCIN3*, in the CIN subclade encode proteins with the R-domain (Fig. 1B). In addition, in Arabidopsis, five of the CIN members are post-transcriptionally regulated by miRNA319 (*AtTCP2*, *3*, *4*, *10*, and *24*) (Palatnik *et al.*, 2003, 2007; Ori *et al.*, 2007). Six orchid TCP genes in the CIN subclade were identified to have a putative binding site for *Phalaenopsis miR319* (Fig. 1C). This finding suggests the regulation of leaf development by a redundant set of miRNA-regulated homologous TCP genes in orchid.

To determine the phylogenetic relationships of *PeTCP* and other *TCP* genes, we reconstructed the phylogeny for the known *TCP* family using the conceptual amino acid sequences of the respective genes as input data. Phylogenetic analysis of the putative protein with TCP proteins from Arabidopsis (*Arabidopsis thaliana*), rice (*Oryza sativa*), and *Phalaenopsis* (*P. equestris*) distinguished two types of TCP proteins based on differences within their TCP domains: class I and class II (Figs 1A and 2). In addition, *PeCIN5* and *PeCIN9* as well as *PeCYC1*, *PeCYC2*, and *PeCYC3* may be duplicated genes in orchid (Fig. 2). Pairwise alignment analysis showed that PeCIN5 and PeCIN9 obtain the lowest *E*-value, 2e–51, compared with those of PeCIN5 or PeCIN9 to other PeCIN proteins. Indeed, *PeCYC2* and *PeCYC3* are located in the same assembled scaffold (Table 1).

### Expression profiles of TCP genes in *P. equestris*

**Fig. 3.**
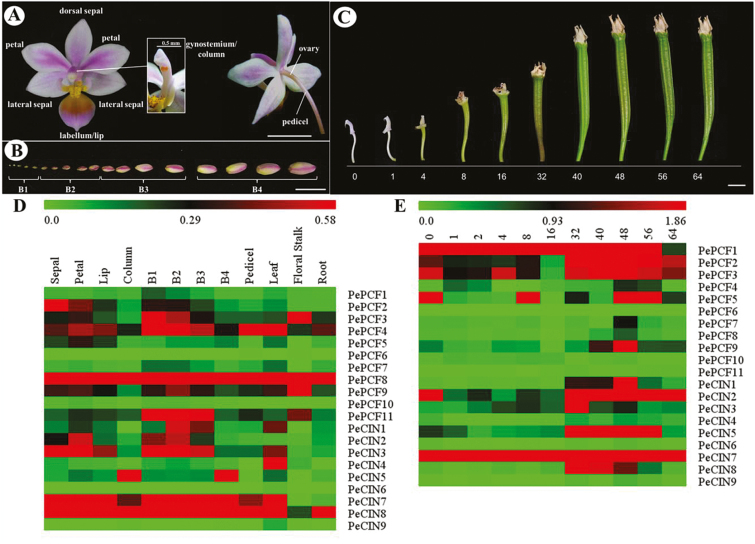
Heat map representation for the expression profiles of *PeTCP* genes. (A) Structure of a *P. equestris* flower. (B) Floral bud development stages from B1 to B4. (C) Morphological changes in the ovary at various days after pollination. Expression patterns of (D) 11 *PePCF* genes and nine *PeCIN* genes in various organs and developmental stages of floral bud; (E) 11 *PePCF* genes and nine *PeCIN* genes in various developmental stages of ovule. *PeActin4* was used as an internal control. The expression levels are represented by the color bar. B1: stage 1 flower bud (0~1mm); B2: stage 2 flower bud (1~2mm); B3: stage 3 flower bud (2~5mm); B4: stage 4 flower bud (5~10mm); Co: column; DAP, days after pollination; F: floral stalk; L: leaf; Li: lip; P: pedicel; Pe: petals; R: root; Se: sepals. Scale bar: 1cm.

**Fig. 4.**
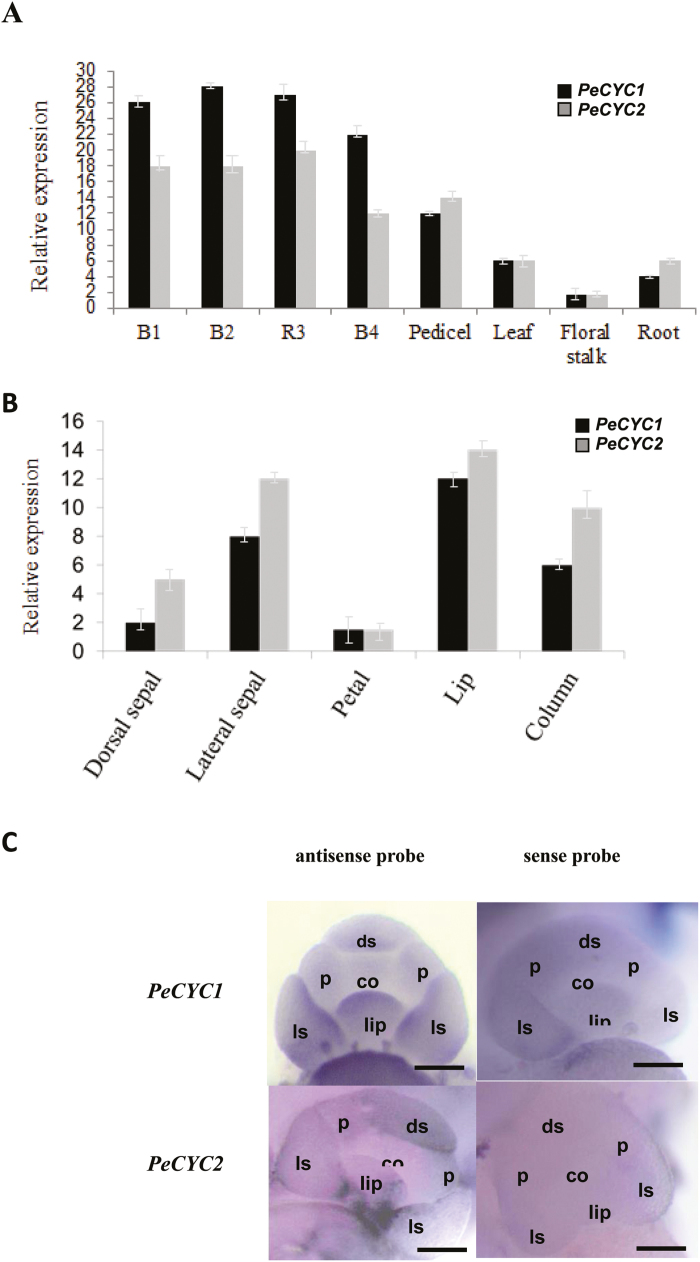
Spatial and temporal expression patterns of *PeCYC*s genes. (A) Real-time quantitative RT-PCR (qRT-PCR) analysis of the mRNA expression of *PeCYC1* and *PeCYC2* in various tissues and developmental stages of floral bud. (B) qRT-PCR analysis of the mRNA expression of *PeCYC1* and *PeCYC2* in different floral organs. (C) Whole mount *in situ* hybridization of *PeCYC*1 and *PeCYC2* in early floral development stage. Upper left: antisense probe of *PeCYC1*; upper right: sense probe of *PeCYC1*; lower left: antisense probe of *PeCYC2*; lower right: sense probe of *PeCYC*2. co: column primordium; ds: dorsal sepal primordium; ls: lateral sepal primordium; p: petal primordium. Scale bar: 100 μm.

We determined the spatial and temporal expression profiles of TCP genes in *P. equestris* of different floral organs (sepal, petal, labellum/lip, and gynostemium/column) (Fig. 3A), various developmental stages of floral buds (B1, 2, 3, and 4) (Fig. 3B; Tsai *et al.*, 2004), pedicel, leaf, floral stalk and root, and different developmental stages of ovule after pollination (Fig. 3C; Chen *et al.*, 2012) by real-time RT-PCR analysis (Fig. 4 and Supplementary Figs S1–S8). The expression patterns of 11 *PePCF*-like and nine *PeCIN*-like genes are shown in Fig. 3. The expression in different tissues varied widely among orchid TCP genes and among different organs for individual TCP genes. The results suggested the functional divergence of orchid genes during plant developmental processes.

Most of the transcripts of *PePCF*-like genes could be detected in vegetative and reproductive tissues. However, *PePCF6* was not expressed in any tissues, and the expression of *PePCF11* was not detected in developing ovules (Fig. 3D, E and Supplementary Fig. S9). Many *PeCIN*-like genes showed wide expression in floral organs, developing floral bud, pedicel, leaf, floral stalk, and root (Fig. 3D and Supplementary Fig. S9A). Four genes (*PeCIN2*, *5*, *7*, and *8*) expressed in developing ovule, especially in the late developmental stage of ovule (40–64 days after pollination [DAP]) (Fig. 3E and Supplementary Fig. S9B). However, the expression of *PeCIN6* was not detected in any tissue (Fig. 3D, E and Supplementary Fig. S9), but *PeCIN9* specifically expressed in leaf (Fig. 3D and Supplementary Fig. S9A).

As compared with the four *PeCIN*-like genes, nine *PePCF* genes were expressed in developing ovule (Fig. 3E and Supplementary Fig. S9B). This may imply that *PePCF*-like genes play more important roles in ovule differentiation and growth. Of note, *PePCF10* predominantly expressed in ovules at early developmental stages (0–16 DAP) and *PeCIN8* showed high expression at late developmental stages in ovule (16–64 DAP) (Fig. 3E and Supplementary Fig. S9B). Transcripts of *PePCF6* and *PeCIN6* were not detected in any organs, so they may be primarily expressed in other organs not tested or under special conditions (Fig. 3D, E and Supplementary Fig. S9).

### *PeCYC* genes were predominantly expressed in developing floral buds

Expression profiles of three *PeCYC*s (*PeCYC1*, *PeCYC2*, and *PeCYC3*) were also analysed by real-time RT-PCR. Results showed that both *PeCYC1* and *PeCYC2* were predominantly expressed in developing floral buds (Fig. 4A). However, the expression of *PeCYC3* was not detected. Expression analysis of *PeCYC1* and *PeCYC2* was further conducted in dissected floral organs (including the dorsal/lateral sepal, petal, lip, and column). Both *PeCYC1* and *PeCYC2* were strongly expressed in the lateral sepal, lip, and column (Fig. 4B). The detailed spatial expression patterns of *PeCYC1* and *PeCYC2* at the early stage of floral development were further investigated by whole mount *in situ* hybridization by antisense RNA probes. Results showed transcripts of *PeCYC1* were detected at the primordia of lateral sepal and lip (Fig. 4C, upper left panel). However, transcripts of *PeCYC2* were concentrated at the dorsal parts of the lateral sepal and lip primordia (Fig. 4C, lower left panel). Expression of *PeCYC*s at the floral dorsal region might correlate with orchid floral zygomorphic development.

### Nuclear localization of class I PePCF10 and class II PeCIN8 proteins

**Fig. 5.**
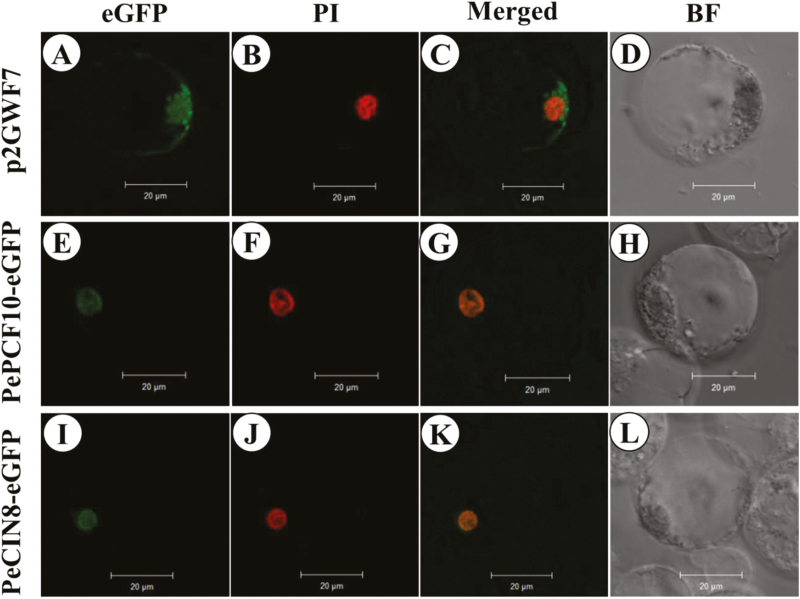
Localization patterns of PePCF10–GFP and PeCIN8–GFP fusion proteins in *P. aphrodite* petal protoplasts. Pictures show fluorescence and bright-field confocal microscopy images. Images were merged with use of Axio Vision Rel.4.8 software. (A) Empty vector green fluorescence in the cytoplasm and nucleus of a flower cell. (B) Cell in (A) stained with propidium iodide (PI; red) to show the nucleus. (C) Merged image of (A) and (B) to show green fluorescence both in the cytoplasm and the nucleus of a flower cell. (D) Cell in (A), (B) and (C) visualized by bright-field confocal microscopy. (E) PePCF10–GFP green fluorescence in the nucleus of a flower cell. (F) Cell in (E) stained with PI (red) to show the nucleus. (G) Merged image of (E) and (F) to show the nuclear localization in a flower cell. (H) Cell in (E), (F) and (G) visualized by bright-field confocal microscopy. (I) PeCIN8–GFP green fluorescence in the nucleus of a flower cell. (J) Cell in (I) stained with PI (red) to show the nucleus. (K) Merged image of (I) and (J) to show the nuclear localization in a flower cell. (L) Cell in (I), (J) and (K) visualized by bright-field confocal microscopy. Scale bars: 20 μm.

Orchid ovule development is precisely initiated by pollination. Because of the successive expression of *PePCF10* and *PeCIN8* during ovule development, we further characterized these two genes. To examine the subcellular localization of the two proteins, PePCF10–GFP and PeCIN8–GFP fusion proteins were constructed, respectively. Both PePCF10–GFP and PeCIN8–GFP fusion proteins were detected predominantly in nuclei derived from *Phalaenopsis* petal protoplasts (Fig. 5). The results are consistent with sequence-based prediction of the subcellular localization and putative transcription factor activity of PePCF10 and PeCIN8.

### Interaction behaviors of class I PePCF10 and class II PeCIN8 proteins

**Fig. 6.**
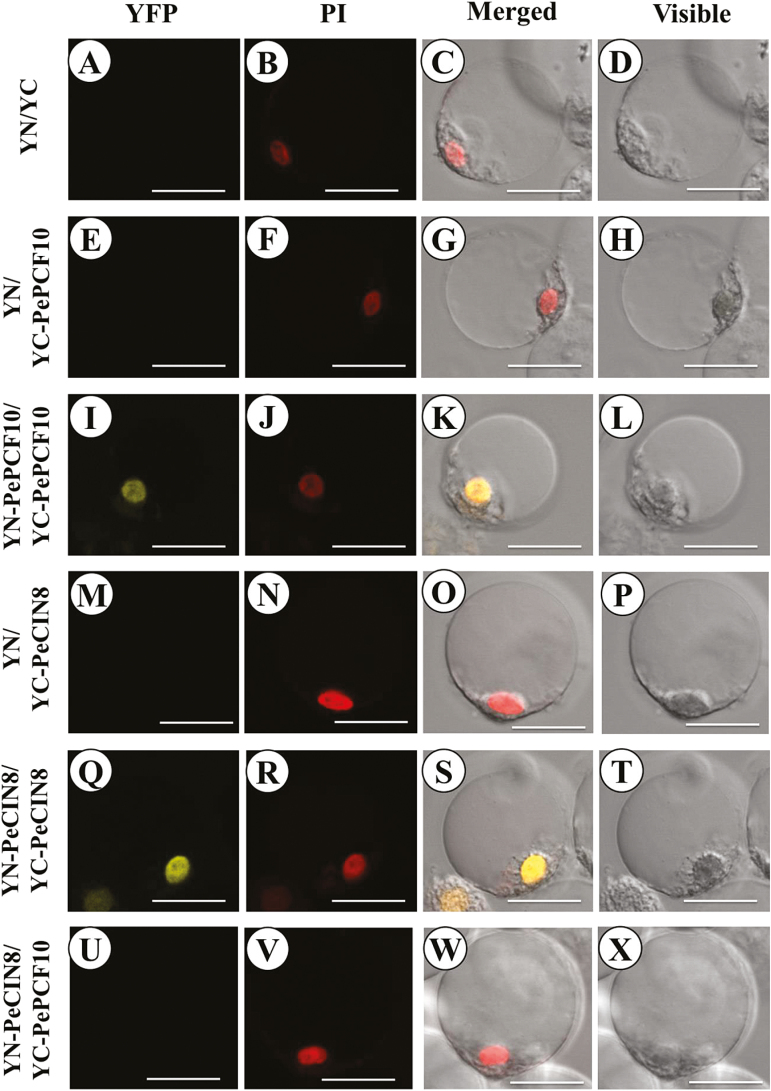
Bimolecular fluorescence complementation (BiFC) visualization of TCP dimerization in transiently transfected *P. aphrodite* petal protoplasts. (A–D) Co-expression of non-fused YN with non-fused YC was unable to reconstitute a fluorescent YFP chromophore. (E–H) Co-expression of non-fused YN with YC-PePCF10 was also unable to reconstitute a fluorescent YFP chromophore. (I–L) PeTCP10 interacts with PeTCP10 protein in nucleus of flower cell. (M–P) Co-expression of non-fused YN with YC-PeCIN8 was also unable to reconstitute a fluorescent YFP chromophore. (Q–T) PeCIN8 interacts with PeCIN8 protein in nucleus of flower cell. (U–X) Co-expression of YN-PeCIN8 with YC-PePCF10 was unable to reconstitute a fluorescent YFP chromophore. The fluorescence indicates interaction between the indicated partner proteins. The images were obtained from the yellow fluorescent protein channel or bright field or are a merged image of the yellow fluorescence, and cells stained with PI represented in red. Scale bars: 20 μm.

Several studies have provided evidence that TCP TFs can bind DNA as homo- or heterodimers. So far TCPs have been found to form heterodimers only between specific members of the same class (Kosugi and Ohashi, 2002; Viola *et al.*, 2011; Danisman *et al.*, 2012; Yang *et al.*, 2012; Aguilar-Martinez and Sinha, 2013). To investigate the protein–protein interactions between PePCF10 and PeCIN8 proteins, we used bimolecular fluorescence complementation (BiFC) assay. The constructs were co-transfected and transiently expressed in the petal protoplasts of *Phalaenopsis*. Interaction fluorescence signals were observed in YN–PePCF10 and YC–PePCF10 (Fig. 6I, J, K, L) as well as YN–PeCIN8 and YC–PeCIN8 (Fig. 6Q, R, S, T), which suggests that both PePCF10 and PeCIN8 can form homodimers. In addition, the signals were localized in the nucleus, as indicated by the nuclear dye propidium iodide (PI). However, interaction was not observed with the combination of YN–PeCIN8 and YC–PCF10 (Fig. 6U, V, W, X). Therefore, PePCF10 and PeCIN8 may not form heterodimers. No fluorescence was detected with the empty vector control (Fig. 6A, B, C, D) and no yellow fluorescence was detected when only the C-terminal half of the YFP was co-bombarded with *PePCF10* or *PeCIN8* constructs (Fig. 6E, F, G, H, M, N, O, P). Yeast two-hybrid experiments were also performed to analyse the behavior of PePCF10 and PeCIN8. The results were similar to those of the BiFC assay, except that PePCF10 homodimer was not observed (Supplementary Fig. S10). It is possible that interaction between PePCF10 proteins was blocked by the steric hindrance of fused PePCF10–GAL4 protein. We also tested interactions between PePCF10 and 23 Arabidopsis TCPs systematically by yeast two-hybrid assay. Results showed that PePCF10 is considered to interact with many TCP proteins which belong to the PCF, CYC/TB1, or CIN clade (Supplementary Fig. S11), suggesting that PePCF10 may have broad specificity in terms of its interacting partners. Arabidopsis TCP14, which is closest to PePCF10, appears to interact with PePCF10 strongly, supporting the above-described BiFC data in which PePCF10 forms a homodimer. However, Arabidopsis TCP3, TCP4, and TCP10, which are close to PeCIN8, also showed interaction with PePCF10 (Supplementary Fig. S11). These data suggest that the specificity of protein–protein interaction of TCP could be somewhat different between Arabidopsis and orchid.

### Functional characterization of the two *PeTCP* genes in transgenic Arabidopsis

**Fig. 7.**
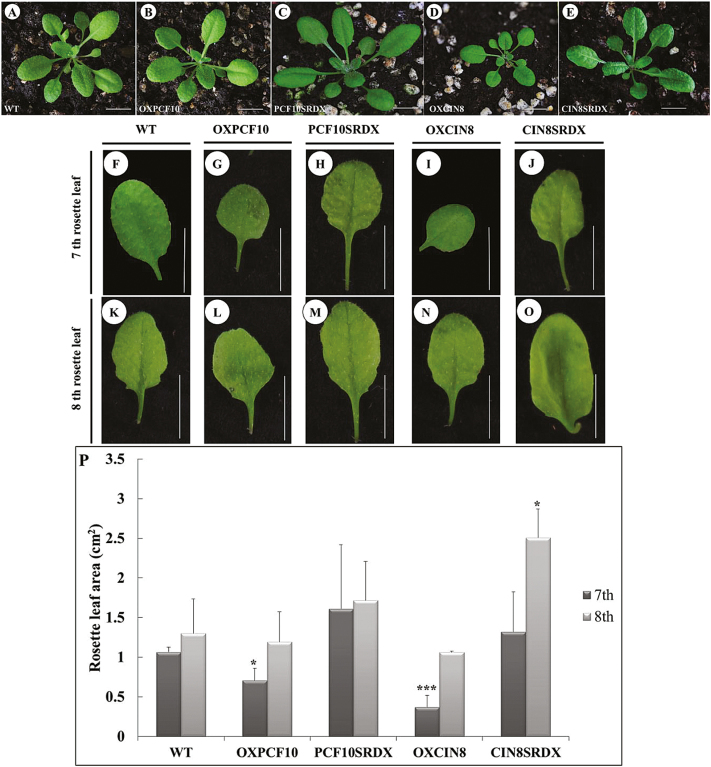
Rosette leaves of wild-type (WT) and transgenic Arabidopsis plants. (A–E) rosette of WT (A), *OXPCF10* (B), *PCF10SRDX* (C), *OXCIN8* (D) and *CIN8SRDX* (E). (F–J) Seventh rosette leaf from WT (F), *OXPCF10* (G), *PCF10SRDX* (H), *OXCIN8* (I) and *CIN8SRDX* (J). (K–O) Eighth rosette leaf from WT (K), *OXPCF10* (L), *PCF10SRDX* (M), *OXCIN8* (N) and *CIN8SRDX* (O). (P) Seventh and eighth rosette leaf area of WT, *OXPCF10*, *PCF10SRDX*, *OXCIN8* and *CIN8SRDX*. Asterisks indicate statistically significant differences (*P<0.05, ***P<0.001 compared with WT by Student’s *t*-test). Errors bar represent the SD of three biological repeats. Scale bars: 1cm.

**Fig. 8.**
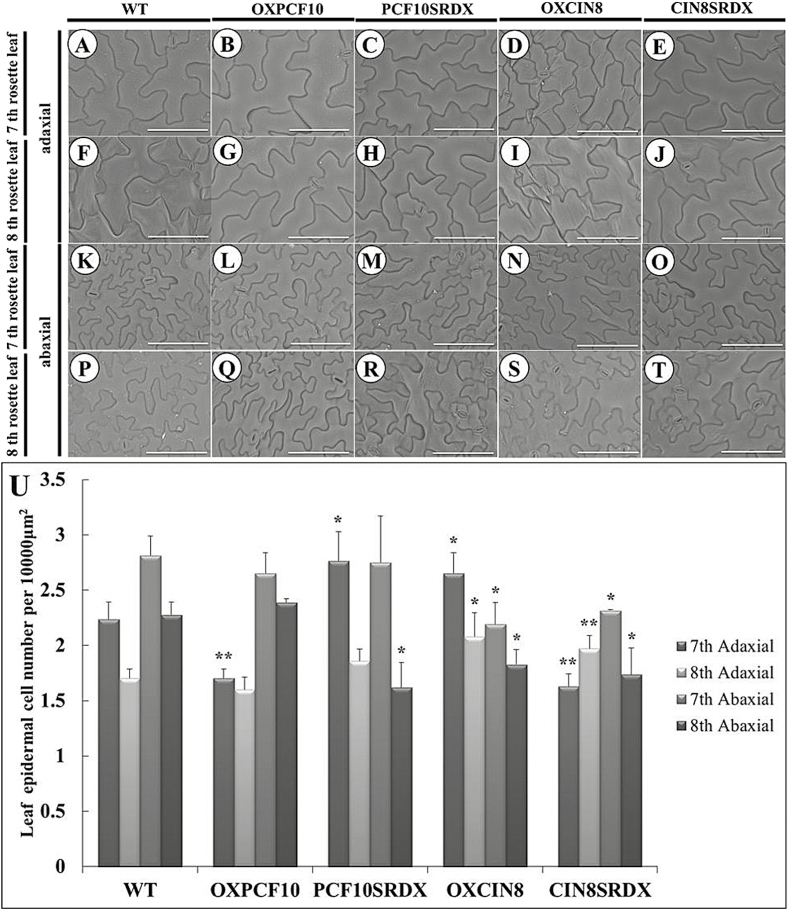
Scanning electron micrographs of leaf epidermal cells of WT and transgenic Arabidopsis plants. (A–E) Adaxial epidermis in the seventh rosette leaf from WT (A), *OXPCF10* (B), *PCF10SRDX* (C), *OXCIN8* (D) and *CIN8SRDX* (E). (F–J) Adaxial epidermis in the eighth rosette leaf from WT (F), *OXPCF10* (G), *PCF10SRDX* (H), *OXCIN8* (I) and *CIN8SRDX* (J). (K–O) Abaxial epidermis in the seventh rosette leaf from WT (K), *OXPCF10* (L), *PCF10SRDX* (M), *OXCIN8* (N) and *CIN8SRDX* (O). (P–T) Abaxial epidermis in the eighth rosette leaf from WT (P), *OXPCF10* (Q), *PCF10SRDX* (R), *OXCIN8* (S) and *CIN8SRDX* (T). (U) Seventh and eighth rosette leaf epidermal cell number for WT, *OXPCF10*, *PCF10SRDX*, *OXCIN8* and *CIN8SRDX*. Asterisks indicate statistically significant differences (*P<0.05, **P<0.01, compared with WT by Student’s *t*-test). Errors bar represent the SD of three biological repeats (*n*=200 each). Scale bars: 100 μm.

We constructed transgenic Arabidopsis plants expressing *PePCF10* (OXPCF10) under the control of the CaMV 35S promoter via Agrobacterium-mediated transformation. Twenty-five of the 100 independently transformed OXPCF10 transgenic T1 lines showed kanamycin resistance and a similar phenotype. Among 25 T2 lines, 12 showed a 3:1 segregating kanamycin resistance phenotype. The rosette size of OXPCF10 plants was normal (Fig. 7B), and we chose the seventh and eighth rosette leaf for observation. OXPCF10 plants showed a round-leaf phenotype (Fig. 7G, L). The leaf area of the seventh rosette leaf was smaller for OXPCF10 than wild-type (WT) plants (Fig. 7P). A previous study indicated that Arabidopsis leaf size regulation is mainly controlled by cell number and size (Tsukaya, 2006). To assess the contributions of cell number and size to the reduced leaf size of the seventh rosette leaf in OXPCF10 plants, we compared the seventh and eighth rosette leaves of WT and transgenic plants by scanning electron microscopy. For seventh rosette leaves, the adaxial epidermal cell size was larger for OXPCF10 than WT plants, and the adaxial epidermal cell number was significantly reduced (Fig. 8A, B, U). For the eighth rosette leaves, the adaxial epidermal cell size and cell number did not differ between WT and transgenic plants (Fig. 8F, G, U); the abaxial epidermal cell size and cell number of seventh rosette leaves (Fig. 8K, L, U) and eighth rosette leaves did not differ (Fig. 8P, Q, U). The altered leaf size and round leaves of OXPCF10 plants were related to decreased adaxial epidermal cell number and increased cell size.

**Fig. 9.**
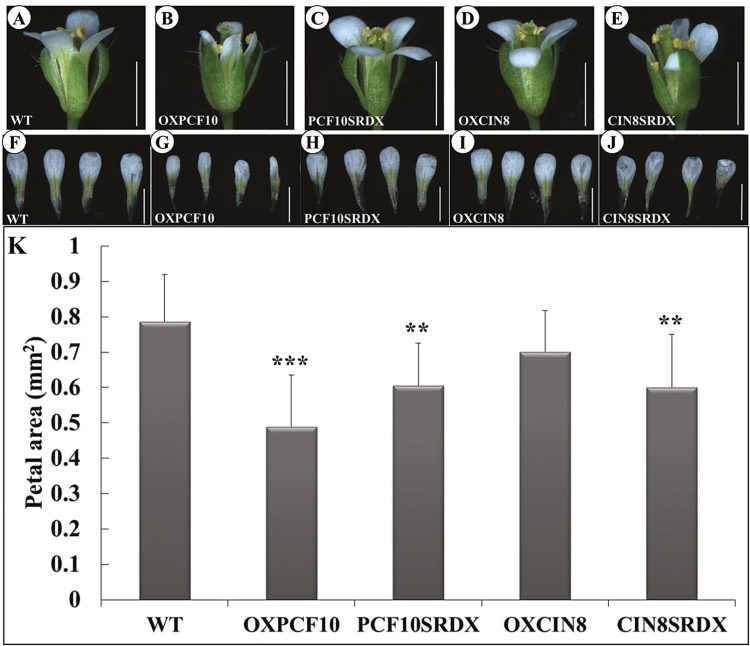
Flowers of WT and transgenic Arabidopsis plants. (A–E) Side views of WT (A), *OXPCF10* (B), *PCF10SRDX* (C), *OXCIN8* (D) and *CIN8SRDX* (E). (F–J) Petals of WT (F), *OXPCF10* (G), *PCF10SRDX* (H), *OXCIN8* (I) and *CIN8SRDX* (J). (K) Petal area of WT, *OXPCF10*, *PCF10SRDX*, *OXCIN8* and *CIN8SRDX*. Asterisks indicate statistically significant differences (**P<0.01, ***P<0.001, compared with WT by Student’s *t*-test). Errors bar represent the SD of three biological repeats (*n*=10 each). Scale bars: 1mm.

**Fig. 10.**
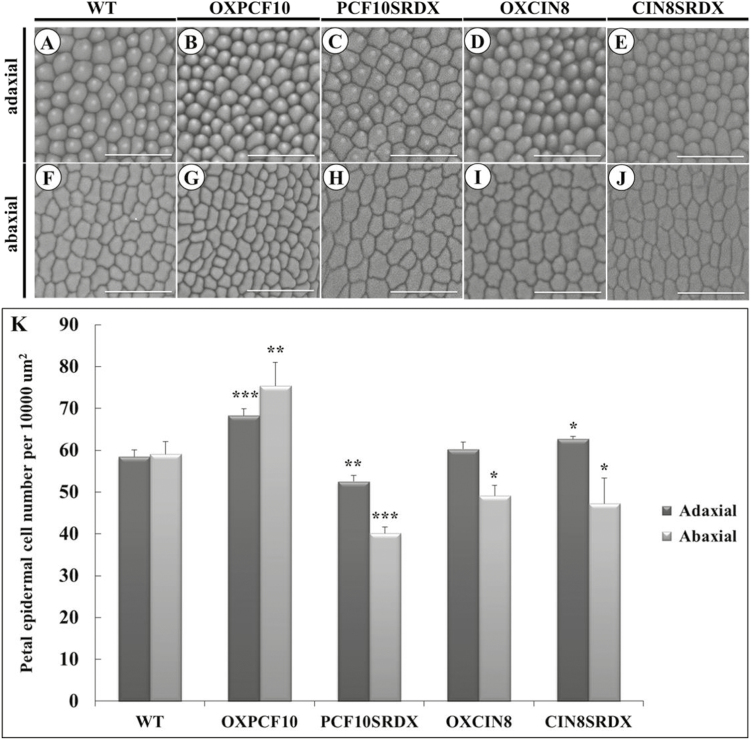
Scanning electron micrographs of epidermal cells of WT and transgenic Arabidopsis petals. (A–E) Adaxial epidermal cells of WT (A), *OXPCF10* (B), *PCF10SRDX* (C), *OXCIN8* (D) and *CIN8SRDX* (E). (F–J) Abaxial epidermal cells of WT (F), *OXPCF10* (G), *PCF10SRDX* (H), *OXCIN8* (I) and *CIN8SRDX* (J). (K) Epidermal cell number of WT, *OXPCF10*, *PCF10SRDX*, *OXCIN8* and *CIN8SRDX*. Asterisks indicate statistically significant differences (*P<0.05, **P<0.01, ***P<0.001, compared with WT by Student’s *t*-test). Errors bar represent the SD of three biological repeats (*n*=200 each). Scale bars: 50 μm.

The flowers were smaller and less expanded for OXPCF10 than WT plants (Fig. 9A, B). Flower petals were smaller (Fig. 9F, G, K) and the adaxial petal epidermis contained cells with reduced size (Fig. 10A, B) but increased number (Fig. 10K). A similar phenotype was observed for abaxial epidermal cells (Fig. 10F, G, K). The altered petal size of OXPCF10 plants was related to the reduced epidermal cell size and increased epidermal cell number.

**Fig. 11.**
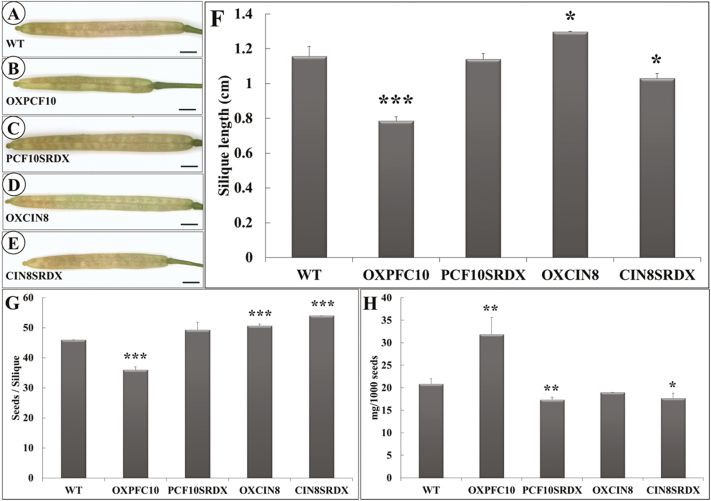
Siliques and seeds of WT and transgenic Arabidopsis plants. (A–E) Siliques of WT (A), *OXPCF10* (B), *PCF10SRDX* (C), *OXCIN8* (D) and *CIN8SRDX* (E). (F) Silique length of WT, *OXPCF10*, *PCF10SRDX*, *OXCIN8* and *CIN8SRDX*. Asterisks indicate statistically significant differences (*P<0.05, ***P<0.001, compared with WT by Student’s *t*-test). Errors bar represent the SD of three biological repeats (*n*=20 each). (G) Seeds per silique of WT, *OXPCF10*, *PCF10SRDX*, *OXCIN8* and *CIN8SRDX*. Asterisks indicate statistically significant differences (***P<0.001, compared with WT by Student’s *t*-test). Errors bar represent the SD of three biological repeats (*n*=20 each). (H) Weight (mg) of 1000 seeds of WT, *OXPCF10*, *PCF10SRDX*, *OXCIN8* and *CIN8SRDX* plants. Asterisks indicate statistically significant differences (*P<0.05, **P<0.01 compared with WT by Student’s *t*-test). Errors bar represent the SD of three biological repeats. Scale bars: 1mm.

Siliques were shorter for OXPCF10 than WT plants (Fig. 11A, B, F). The total seed number per silique of OXPCF10 plants was significantly reduced (Fig. 11G). Big reduction of seeds observed in the overexpressed plants might relate to the abnormal gametogenesis (Takeda *et al.*, 2006; Sarvepalli and Nath, 2011; Viola *et al.*, 2011). The seed size was larger for OXPCF10 than WT plants (data not shown) and the seed weight was significantly increased (Fig. 11H). The altered silique length and seed size and weight of OXPCF10 plants suggested that *PePCF10* may be involved in reproductive development.

In addition to overexpression of *PePCF10* in Arabidopsis, we converted PePCF10 to a chimeric repressor by fusing it with the SRDX repression domain (PCF10SRDX). Twenty-six of the 100 independently transformed PCF10SRDX transgenic T1 lines showed kanamycin resistance and a similar phenotype. Among 26 T2 lines, 13 showed a 3:1 segregating kanamycin resistance phenotype. The rosette size of PCF10SRDX plants was reduced and leaves showed a wrinkled surface and downward curl (Fig. 7C). The leaf area of the seventh and eighth rosette leaf did not differ between WT and transgenic plants, but transgenic lines showed slightly enlarged leaves (Fig. 7F, H, K, M, P). Similar phenotypes were reported in mutant and transgenic lines, with altered proliferation of leaf blade cells (Serrano-Cartagena *et al.*, 2000; Bemis and Torii, 2007; Kieffer *et al.*, 2011). For seventh rosette leaves, the adaxial epidermis contained cells with reduced size but increased number in PCF10SRDX plants (Fig. 8A, C, U). For eighth rosette leaves, the adaxial epidermal cell size and cell number did not differ between WT and transgenic plants (Fig. 8F, H, U) and the abaxial epidermal cell size and cell number of seventh rosette leaves did not differ (Fig. 8K, M, U). The abaxial epidermal cell size of the eighth rosette leaves was larger for transgenic than WT plants and the cell number was less (Fig. 8P, R, U). Although the leaf area was not altered in PCF10SRDX plants (Fig. 7P), the adaxial epidermal cell size was smaller and cell number greater in the seventh rosette leaf and the abaxial epidermal cell size was larger and cell number lower in the eighth rosette leaf. *PePCF10* may regulate cell proliferation and differentiation and thus generate a wrinkled surface and downwardly curled rosette leaves.

The morphology of the flowers did not differ between PCF10SRDX and WT plants (Fig. 9A, C). However, petals were smaller for PCF10SRDX than WT flowers (Fig. 9H, K). The adaxial epidermal cell size of petals did not differ (Fig. 10A, C), but the adaxial epidermal cell number was lower (Fig. 10K). In addition, the abaxial epidermal cell size of petals was larger for PCF10SRDX than WT plants (Fig. 10F, H) and the abaxial epidermal cell number was less (Fig. 10K). The silique length, total seed number per silique, and seed size did not differ between the WT and PCF10SRDX plants (Fig. 11A, C, F, G), but the seed weight of PCF10SRDX plants was significantly decreased (Fig. 11H). These results suggest a function for *PePCF10* in cell proliferation in vegetative and reproductive tissues.

The same strategy was adopted to functionally characterize *PeCIN8*. Twenty-four of the 100 independently transformed OXCIN8 transgenic T1 lines showed kanamycin resistance and a similar phenotype. Among 24 T2 lines, 11 showed a 3:1 segregating kanamycin resistance phenotype. The rosette size of OXCIN8 plants was reduced (Fig. 7D). The rosette leaves showed a round-leaf phenotype (Fig. 7I, N). The leaf area of the seventh rosette leaf was smaller compared with the WT plants. The leaf area of the eighth rosette leaf did not differ when compared with WT (Fig. 7P). Similar phenotypic effects were observed on transgenic lines with altered proliferation of leaf blade cells (Schommer *et al.*, 2008; Sarvepalli and Nath, 2011). The adaxial epidermal cell size of the seventh and eighth rosette leaf was smaller for OXCIN8 than WT plants (Fig. 8A, D, F, I) and the adaxial epidermal cell number was greater (Fig. 8U). The abaxial epidermal cells of the seventh and eighth rosette leaf of OXCIN8 plants showed the opposite pattern. The abaxial epidermal cell size of the seventh and eighth rosette leaf was larger for OXCIN8 than WT plants (Fig. 8K, N, P, S) and the abaxial epidermal cell number was less (Fig. 8U). The altered leaf size and round leaves of OXCIN8 plants were related to changed cell number and cell size in both the adaxial and abaxial sides of the leaf.

The adaxial epidermal cell size and cell number did not differ between OXCIN8 and WT petals (Fig. 10A, D, K). However, the abaxial epidermal cell size was larger for OXCIN8 than WT (Fig. 10F, I) and the abaxial epidermal cell number was less (Fig. 10K). Although the petal area was not modified in OXCIN8 plants (Fig. 9K), the abaxial epidermal cell size was larger and cell number lower than those of WT petals. The siliques were longer for OXCIN8 than WT plants (Fig. 11A, D, F). The total seed number per silique was increased in OXCIN8 plants (Fig. 11G). However, the seed size did not differ between OXCIN8 and WT seeds and seed weight did not differ (Fig. 11H).

*PeCIN8* fused with SRDX-overexpressed transgenic plants (CIN8SRDX) were also produced. Twenty-five of the 100 independently transformed CIN8SRDX transgenic T1 lines showed kanamycin resistance and a similar phenotype. Among 25 T2 lines, 12 showed a 3:1 segregating kanamycin resistance phenotype. The rosette size of CIN8SRDX plants was normal (Fig. 7E). However, the rosette leaves showed a wrinkled surface and a downward curl (Fig. 7E, J, O). The leaf area of the seventh rosette leaf seemed slightly larger for CIN8SRDX than WT plants; however, the eighth rosette leaf area was significantly larger (Fig. 7P). For seventh rosette leaves, the adaxial epidermal cell size was larger for CIN8SRDX than WT plants, and the adaxial epidermal cell number was smaller (Fig. 8A, E, U). For eighth rosette leaves, the adaxial epidermal cell size seemed smaller for CIN8SRDX than WT plants and the adaxial epidermal cell number was higher (Fig. 8F, J, U). For both seventh and eighth rosette leaves, the abaxial epidermal cell size was larger for *CIN8SRDX* than WT plants and the abaxial epidermal cell number was lower (Fig. 8K, O, P, T, U). These results suggest that the wrinkled surface and downwardly curled rosette leaves of CIN8SRDX plants were due to increased adaxial epidermal cell number and abaxial epidermal cell size.

The flowers of CIN8SRDX plants were less expanded (Fig. 9E) and significantly smaller than those of WT plants (Fig. 9F, J, K). The adaxial epidermal cell size seemed smaller for CIN8SRDX than WT petals (Fig. 10A, E) and the adaxial epidermal cell number was significantly increased (Fig. 10K). The abaxial epidermal cells were larger for CIN8SRDX than WT petals because of elongated cells (Fig. 10F, J), and the abaxial epidermal cell number was significantly decreased (Fig. 10K). The siliques were shorter for CIN8SRDX than WT plants (Fig. 11A, E, F) and the total seed number per silique was significantly increased (Fig. 11G). The seed weight was greater for CIN8SRDX than WT plants (Fig. 11H).

## Discussion

In the present study, we identified 23 TCP genes from *P. equestris*. The number of TCP genes in *P. equestris* was similar to that in Arabidopsis (24) and rice (22) (Martín-Trillo and Cubas, 2010). As well, the gene number in each subfamily of TCP genes was similar among the three species (Fig. 2). Although the genome size of *Phalaenopsis* is larger than that of Arabidopsis and rice—approximately 9 and 2.8 times, respectively—the number of genes among the genomes is similar (Cai *et al.*, 2015). This fact suggests that the TCP family did not expand along with genome expansion of *P. equestris* during orchid evolution (Cai *et al.*, 2015). TCP genes in orchids, like those Arabidopsis, rice and other plants, may participate in regulating multiple aspects of plant development such as gametophyte development (Takeda *et al.*, 2006; Sarvepalli and Nath, 2011), hormone signal transduction (Aguilar-Martinez *et al.*, 2007; Guo *et al.*, 2010; Yanai *et al.*, 2011), mitochondria biogenesis (Hammani *et al.*, 2011), regulation of circadian clock (Pruneda-Paz *et al.*, 2009; Giraud *et al.*, 2010), lateral branching (Takeda *et al.*, 2003; Aguilar-Martinez *et al.*, 2007), flower development (Koyama *et al.*, 2011; Uberti-Manassero *et al.*, 2012), seed germination (Tatematsu *et al.*, 2008; Rueda-Romero *et al.*, 2012), and leaf development (Kieffer *et al.*, 2011). Actually, the expression of TCP genes was broadly detected in various vegetative, reproductive tissues and different developmental stages of *Phalaenopsis* orchid (Fig. 3).

In most orchid flowers, ovary and ovule development is precisely triggered by pollination (Tsai *et al.*, 2008). Auxin is a positive regulator for the orchid ovule initiation (Tsai *et al.*, 2005; Novak *et al.*, 2014). We found *PePCF10* predominantly expressed in ovules at early developmental stages (0–16 DAP) and *PeCIN8* showed high expression at late developmental stages in ovules (16–64 DAP) (Fig. 3E and Supplementary Fig. S9B), with overlapping expression at 16 DAP. At about 16 DAP, when *PePCF10* and *PeCIN8* are co-expressed, placental protuberances develop from a single epidermal layer of the placenta (Chen *et al.*, 2012). Archesporial cells are differentiated at the terminus of the nucellar filament. Later, periclinal divisions in epidermal cells surrounding the archesporial cells generate the inner integument (Arditti and Pridgeon, 1997). Thus, the function of the homodimer TF PePCF10 might be related to placenta growth and continued initiation of archesporial cells and inner integument. In addition, a homodimer of PeCIN8 might associate with the later developmental process of megagametogenesis. Further study of the relationship between *PePCF10* and *PeCIN8* will allow us to gain new insights into the regulation of the unique system of orchid ovule development.

Functional analysis by overexpressing *PePCF10* or a chimeric repressor gene in Arabidopsis suggested that *PePCF10* plays a role in regulating epidermal cell proliferation and differentiation. Previous study suggested that TCP proteins could be dual-function transcription factors able to positively or negatively regulate expression of target genes (Herve *et al.*, 2009; Wang *et al.*, 2013). Although the seventh and eighth rosette leaf areas of PCF10SRDX lines slightly differed from those of the WT, the epidermal cell number was increased or decreased in PCF10SRDX lines depending on the different epidermal cell axis. *PePCF10* may act as a dual functional TF in regulating cell proliferation in the developing leaf, and the distinctive activities of PePCF10 may depend on its interaction with other proteins in the adaxial or abaxial side of the leaf. Our results are consistent with the function of TCP proteins being dependent on the organ, tissue, or cellular context (Herve *et al.*, 2009).

The class II TCP gene *CIN* was reported to act as a repressor of cell proliferation in leaves. High levels of miR319 or low miR319-targeted *TCP* activity might cause excess cell proliferation, thus resulting in a crinkled leaf in Arabidopsis, snapdragon, and tomato or larger leaves in monocotyledonous plants (rice and creeping bentgrass) (Nath *et al.*, 2003; Palatnik *et al.*, 2003; Ori *et al.*, 2007; Yang *et al.*, 2013). In this study, we found that the expression of *PeCIN8* was broadly distributed in orchid vegetative and reproductive tissues (Fig. 3). Overexpressed *PeCIN8* or Arabidopsis with the chimeric repressor gene showed a varied leaf morphology associated with altered cell number and cell size of epidermal cells. In addition, the function of *PeCIN8* was exhibited by the petal size. Furthermore, the epidermal cell number in the affected organs of transgenic plants was increased or decreased depending on different epidermal cell axis, so *PeCIN8* may act as a dual-function TF in regulating cell proliferation in the developing leaf or petal.

Previous studies have reported that Arabidopsis *TCP* genes including *TCP2*, *TCP3*, *TCP4*, *TCP10*, and *TCP24* possess *miR319*-binding sites. The cleavage activity of *miR319* on these *AtTCP* transcripts has been demonstrated (Nag *et al.*, 2009; Palatnik *et al.*, 2003). A similar finding was later shown in tomato *LANCEOLATE* (*LA*, *SlTCP2*), the closest homolog of *AtTCP4* (Ori *et al.*, 2007). Although *PeCIN8* was predicted to possess an *miR319* targeting site, it seems that cleavage of *PeCIN8* transcripts did not occur in the *PeCIN8*-overexpressing Arabidopsis. Koyama *et al.* (2007) reported that the ectopic expression of *AtTCP3* under the control of CaMV 35S promoter (*35S:AtTCP3*) did not show visible abnormalities compared with Arabidopsis wild-type. However, overexpression of a mutant form of *AtTCP3* without the *miR319*-binding sequence (*35S:mAtTCP3*) induced fusion of cotyledons, defects in the formation of shoots and elongation of hypocotyls in Arabidopsis. Similar results were found when *miR319*-resistant or -sensitive versions of *AtTCP2* and *AtTCP4* were expressed, suggesting the phenotype differences are likely due to the presence of *miR319* (Palatnik *et al.*, 2003, 2007). In this study, overexpression of wild-type form of *PeCIN8* induces small rosette size and rounder leaves, indicating a higher level of *PeCIN8* transcripts in the transgenic Arabidopsis plants. These data, which contradict previous results, might be due to no cleavage of the *PeCIN8* transcripts occurring or a lower sensitivity of *PeCIN8* transcripts to Arabidopsis *miR319* regulation. It was reported that only one point mutation at the *miR319*-binding site of tomato *LA* alleles could confer partial resistance of *LA* transcripts to *miR319* cleavage activity (Ori *et al.*, 2007). This dominant mutation of *LA* in tomato resulted in small simple leaves instead of the large compound ones of wild type. In addition, the *AtTCP4* hyper-activation lines that expressed *35S:AtTCP4:VP16* under Arabidopsis Col-0 background show much smaller leaf sizes and rounder leaves (Sarvepalli and Nath, 2011). This suggests that the regulation of TCP genes for plant development depends on the *miR319* level or the ratio of *TCP* transcripts and *miR319*. Furthermore, we could not exclude the possibility that the post-transcriptional regulation of *CIN*-type *TCP* genes is different between Arabidopsis and *P. equestris*.

In conclusion, we identified 23 members of the orchid TCP TF gene family. These genes have broad expression profiles corresponding to multiple aspects of growth and development. Orchid *PePCF10* and *PeCIN8* showed successive expression patterns during ovule development. Additionally, both proteins could form homodimers and localize in the nucleus. Functional analysis suggested that both genes might regulate cell proliferation. These two genes may contribute to the unique functions of TCP TFs in orchids, controlling ovule initiation and development.

## Supplementary data

Supplementary data are available at *JXB* online.

Table S1. Primers used in this study.

Figure S1. The expression patterns of *PePCF* genes in various organs and developmental stages of floral bud of *Phalaenopsis equestris* by real-time RT-PCR analysis. Quantification was normalized to *Actin4*.

Figure S2. The expression patterns of *PePCF* genes in various developmental stages of ovule of *P. equestris* by real-time RT-PCR analysis. Quantification was normalized to *Actin4*.

Figure S3. The expression patterns of *PeCIN* genes in various organs and developmental stages of floral bud of *P. equestris* by real-time RT-PCR analysis with use of *PeActin4* as internal control.

Figure S4. The expression patterns of *PeCIN* genes in various developmental stages of ovule of *P. equestris* by real-time RT-PCR analysis with use of *PeActin4* as internal control.

Figure S5. The expression patterns of *PePCF* genes in various organs and developmental stages of floral bud of *P. equestris* by real-time RT-PCR analysis with use of *Pa18S rRNA* as internal control.

Figure S6. The expression patterns of *PePCF* genes in various developmental stages of ovule of *P. equestris* by real-time RT-PCR analysis with use of *Pa18S rRNA* as internal control.

Figure S7. The expression patterns of *PeCIN* genes in various organs and developmental stages of floral bud of *P. equestris* by real-time analysis with use of *Pa18S rRNA* as internal control.

Figure S8. The expression patterns of *PeCIN* genes in various developmental stages of ovule of *P. equestris* by real-time RT-PCR analysis with use of *Pa18S rRNA* as internal control.

Figure S9. Heat map representation for the expression profiles of *PePCF* and *PeCIN* in various organs and developmental stages of floral bud (A), and in various developmental stages of ovule (B).

Figure S10. Analysis of protein interactions between PePCF10 and PeCIN8 by quantitative *β*-galactosidase activity assays.

Figure S11. Result of yeast two-hybrid assay between PePCF10 and Arabidopsis TCPs (A). Matrix describing experimental design shown in (B) and (C).

Supplementary Data

## Abbreviations:

BiFC: bimolecular fluorescence complementation
CaMV: cauliflower mosaic virus
CYC: CYCLOIDEA
DAP: days after pollination
GFP: green florescent protein
HMM: hidden Markov model
ORF: open reading frame
OX: overexpressing
PCF: proliferating cell factor
TB1: TEOSINTE BRANCHED1
TCP: TEOSINTE-BRANCHED/CYCLOIDEA/PCF
TF: transcription factor
PI: propidium iodide
RT-PCR: reverse transcription–PCR
WT: wild-type
YC: C-terminal half of YFP
YFP: yellow florescent protein
YN: N-terminal half of YFP.
