# Supplementary material for: Genome-wide identification and characterization of TCP genes involved in ovule development of Phalaenopsis equestris
Source: J Exp Bot. 2016 Aug 19;67(17):5051–66. doi: 10.1093/jxb/erw273 (PMC5014156; doi:10.1093/jxb/erw273)
Supplement: Supplementary Data [file supp_67_17_5051__index.html]

Genome-wide identification and characterization of TCP genes involved in ovule development of Phalaenopsis equestris — Genome-wide identification and characterization of TCP genes involved in ovule development of Phalaenopsis equestris — Supplementary Data 

# Genome-wide identification and characterization of *TCP* genes involved in ovule development of *Phalaenopsis equestris*

## Supplementary Data

Data files

- Supplementary\_Figure\_1\_11\_Table\_1.pdf - Supplementary Data
