# Supplementary material for: Genome-wide identification and characterization of TCP genes involved in ovule development of Phalaenopsis equestris
Source: J Exp Bot. 2016 Aug 19;67(17):5051–66. doi: 10.1093/jxb/erw273 (PMC5014156; doi:10.1093/jxb/erw273)
Supplement: Supplementary Data [file supp_erw273_Supplementary_Figure_1_11_Table_1.pdf]

Supporting information figure legends

Figure S1. The expression patterns of *PePCF* genes in various organs and developmental stages of floral bud of *Phalaenopsis equestris* by real-time RT-PCR analysis. Quantification was normalized to *Actin4* for each organ. B1: stage 1 flower bud (0~1 mm); B2: stage 2 flower bud (1~2 mm); B3: stage 3 flower bud (2~5 mm); B4: stage 4 flower bud (5~10 mm). Error bars: ± SD (n=3 each).

Figure S2. The expression patterns of *PePCF* genes in various developmental stages of ovule of *P. equestris* by real-time RT-PCR analysis. Quantification was normalized to *Actin4* for each organ. DAP, days after pollination. Error bars: ± SD (n=3 each).

Figure S3. The expression patterns of *PeCIN* genes in various organs and developmental stages of floral bud of *P. equestris* by real-time RT-PCR analysis. Quantification was normalized to *Actin4* for each organ. B1: stage 1 flower bud (0~1 mm); B2: stage 2 flower bud (1~2 mm); B3: stage 3 flower bud (2~5 mm); B4: stage 4 flower bud (5~10 mm). Error bars: ± SD (n=3 each).

Figure S4. The expression patterns of *PeCIN* genes in various developmental stages of ovule of *P. equestris* by real-time RT-PCR analysis. Quantification was normalized to *Actin4* for each organ. DAP, days after pollination. Error bars: ± SD (n=3 each).

Figure S5. The expression patterns of *PePCF* genes in various organs and developmental stages of floral bud of *P. equestris* by real-time RT-PCR analysis. Quantification was normalized to *18S rRNA* for each organ. B1: stage 1 flower bud (0~1 mm); B2: stage 2 flower bud (1~2 mm); B3: stage 3 flower bud (2~5 mm); B4: stage 4 flower bud (5~10 mm). Error bars: ± SD (n=3 each).

Figure S6. The expression patterns of *PePCF* genes in various developmental stages of ovule of *P. equestris* by real-time RT-PCR analysis. Quantification was normalized to *18S rRNA* for each organ. DAP, days after pollination. Error bars: ± SD (n=3 each).

Figure S7. The expression patterns of *PeCIN* genes in various organs and developmental stages of floral bud of *P. equestris* by real-time RT-PCR analysis. Quantification was normalized to *18S rRNA* for each organ. B1: stage 1 flower bud (0~1 mm); B2: stage 2 flower bud (1~2 mm); B3: stage 3 flower bud (2~5 mm); B4: stage 4 flower bud (5~10 mm). Error bars: ± SD (n=3 each).

Figure S8. The expression patterns of *PeCIN* genes in various developmental stages of ovule of *P. equestris* by real-time RT-PCR analysis. Quantification was normalized to *18S rRNA* for each organ. DAP, days after pollination. Error bars: ± SD (n=3 each).

Figure S9. Heat map representation for the expression profiles of *PeTCP* genes. Expression patterns of (A) 11 *PePCF* genes and 9 *PeCIN* genes in various organs and developmental stages of floral bud; (B) 11 *PePCF* genes and 9 *PeCIN* genes in various developmental stages of ovule. *18S rRNA* was used as an internal control. The expression levels are represented by the color bar. Se: sepals; Pe: petals; Li: lip; Co: column; P: pedicel; F: floral stalk; L: leaf; R: root; B1: stage 1 flower bud (0~1 mm); B2: stage 2 flower bud (1~2 mm); B3: stage 3 flower bud (2~5 mm); B4: stage 4 flower bud (5~10 mm). DAP, days after pollination.

Figure S10. Analysis of protein interactions between TCP proteins.

Quantitative *β*-galactosidase activity assays measuring the interaction of the PePCF10 with PePCF10, PeCIN8 with PeCIN8 and PePCF10 with PeCIN8 proteins. PePCF10 and PeCIN8 were used as bait and expressed as a fusion to the GAL4-BD, as well as both of them were used as prey and expressed as fusion proteins to the GAL4-AD. White bars represent controls. Data are means ± SD of three independent experiments.

Figure S11. Result of yeast two-hybrid assay between PePCF10 and Arabidopsis TCPs

(A) Matrix describing experimental design shown in (B) and (C). Number indicates gene number of Arabidopsis TCPs. For instance, “24” means TCP24. Red, blue, and light blue color indicate strong, intermediate, and weak positive, respectively. (B) Yeast grown on positive-control media lacking leucine and tryptophan. (C) Yeast grown on test media lacking histidine, leucine and tryptophan.

Table S1. Primers used in this study.

Primer Name	Sequence
**Used for real-time RT-PCR analysis**	
PePCF1 real-time RT-PCR F	5'-GTGGTAGTCAGCGATGGACAAG-3'
PePCF1 real-time RT-PCR R	5'-GCCGCCTCCCTCATCAA-3'
PePCF2 real-time RT-PCR F	5'-TGGAGGGTTTATGCAGAGGATT-3'
PePCF2 real-time RT-PCR R	5'-TTGAAGCAAAGCTCATCTGACCTA-3'
PePCF3 real-time RT-PCR F	5'-CCTTCTTCGTTATGGACGTTTCC-3'
PePCF3 real-time RT-PCR R	5'-CCACCCATAGCTCGACCAAA-3'
PePCF4 real-time RT-PCR F	5'-GCTGAGCCGGCCATCA-3'
PePCF4 real-time RT-PCR R	5'-ATTTTGAGAGTTCCGTTGACATTG-3'
PePCF5 real-time RT-PCR F	5'-TATGGCCACCAGCTGTTCCT-3'
PePCF5 real-time RT-PCR R	5'-AGGCCCTTCACTGCTTCCA-3'
PePCF6 real-time RT-PCR F	5'-GACAACGAGCTCATTGCAACA-3'
PePCF6 real-time RT-PCR R	5'-ATCGTTGCCGTCGAAGATG-3'
PePCF7 real-time RT-PCR F	5'-CAGTTTTATATGCAGATGGGATATGG-3'
PePCF7 real-time RT-PCR R	5'-ACTTCATGGCCCAGCTTACAA-3'
PePCF8 real-time RT-PCR F	5'-TCCTCGCCTTCTACTTTCATTCTC-3'
PePCF8 real-time RT-PCR R	5'-TTTCCCACGGACTCCTCCTT-3'
PePCF9 real-time RT-PCR F	5'-GAGCTCTGGTGACGAAGAAAGC-3'
PePCF9 real-time RT-PCR R	5'-TGATCGGCATCCTGATCCTT-3'
PePCF10 real-time RT-PCR F	5'-CCATCCACCAGCACCAACA-3'
PePCF10 real-time RT-PCR R	5'-TTTGGTTGAGCTGCGTTTTG-3'
PePCF11 real-time RT-PCR F	5'-CCCACCGGTTACTTTCAGTTCA-3'
PePCF11 real-time RT-PCR R	5'-TCGGACATCGCGGAGAA-3'
PeCIN1 real-time RT-PCR F	5'-CAGGATGCTACAGCCCATCA-3'
PeCIN1 real-time RT-PCR R	5'-GTGGTCGATCCTGCGGTAAG-3'
PeCIN2 real-time RT-PCR F	5'-AGGCATTGGAAAGGAGAAGGAT-3'
PeCIN2 real-time RT-PCR R	5'-GAAGCACAGTGAAGGAGCTCTGT-3'
PeCIN3 real-time RT-PCR F	5'-GGACCCTTCAGTCCAATCCA-3'
PeCIN3 real-time RT-PCR R	5'-GAAAGGCACGTTTTGCACATC-3'
PeCIN4 real-time RT-PCR F	5'-AGCTCCTGCAGCATTGTTTTCT-3'
PeCIN4 real-time RT-PCR R	5'-GATTTTGCTGAGCTGAGATTCCA-3'
PeCIN5 real-time RT-PCR F	5'-GCATCCAAACTCCAAGCACAT-3'
PeCIN5 real-time RT-PCR R	5'-TTAAAGTTGATGCTCGAAGAATTCC-3'
PeCIN6 real-time RT-PCR F	5'-TTCCAAAGCGCCACAACTG-3'
PeCIN6 real-time RT-PCR R	5'-CCGTGCTTTCCACTCCTAAAAG-3'
PeCIN7 real-time RT-PCR F	5'-AAGGTGGCTCCGGAGTTTATG-3'
PeCIN7 real-time RT-PCR R	5'-GGGTCCCCTCTGTGTTGAAA-3'
PeCIN8 real-time RT-PCR F	5'-GGATGGAGACTATGGATCAGGAA-3'
PeCIN8 real-time RT-PCR R	5'-CCTAGCAGGGATGCGAAAAC-3'
PeCIN9 real-time RT-PCR F	5'-TGGCATACTCTTTTTTTCAGCAAT-3'
PeCIN9 real-time RT-PCR R	5'-TGATGAAGCAAATAGAAAACCTTGA-3'
PeCYC1 real-time RT-PCR F	5'-CAAAGAGGACAACGGCTATGC-3'
PeCYC1 real-time RT-PCR R	5'-GAAAGAACCTTGGAGAAGAAAAAGAAG-3'
PeCYC2 real-time RT-PCR F	5'-GCTAATGAGCTCTATTACAATGCCGAAAAGCAGG-3'
PeCYC2 real-time RT-PCR R	5'-CTCTCTTGATTCTCTGGCAACTAATTTAGACTGAAC-3'
PeCYC3 real-time RT-PCR F	5'-GCTAGCGGCGCTGAAGCAGGTGCTCCG-3'
PeCYC3 real-time RT-PCR R	5'-GGCTGAGAAATGGGCCTCGACCTCAAAATCCTAC-3'
**Used for whole mount in situ hybridization**	
PeCYC1FS	5'-TGCGGAAAACAATCCTCCAGAGCCATCACAACTGC-3'
PeCYC1RS	5'-ATTGCATACGATCCATCGCCGCTGAGCTCCTCATG-3'
PeCYC2FS	5'- GCTAATGAGCTCTATTACAATGCCGAAAAGCAGG-3'
PeCYC2RS	5'-CTCTCTTGATTCTCTGGCAACTAATTTAGACTGAAC-3'
**Used for subcellular localization and BiFC assay**	
PePCF10 attB1 F	5'-GGGGACAAGTTTGTACAAAAAAGCAGGCTTCATGGAAGGAGAAAACATTGGA-3'
PePCF10 attB2 R	5'-GGGGACCACTTTGTACAAGAAAGCTGGGTCGGAGTCGCTGGTGCTCAT-3'
PeCIN8 attB1 F	5'-GGGGACAAGTTTGTACAAAAAAGCAGGCTTCATGAAATCCTCCGGCGAGAT-3'
PeCIN8 attB2 R	5'-GGGGACCACTTTGTACAAGAAAGCTGGGTCGTGATACGAACTAGAGGAA-3'
**Used for ectopic overexpression in *Arabidopsis***	
Full-PePCF10 F	5'-TCTAGAATGGAAGGAGAAAACATTGGAGGAGGA-3'
Full-PePCF10 R	5'-TCTAGACTAGGAGTCGCTGGTGCTCATCGTATC-3'
Full-PeCIN8 F	5'-CCCGGGTATGAAATCCTCCGGCGAGATCTTG-3'
Full-PeCIN8 R	5'-CCCGGGCCTAGTGATACGAACTAGAGGAACGAGAAGACA-3'
**Used for ectopic repression in *Arabidopsis***	
PePCF10-EAR F	5'-CCCGGGCATGGAAGGAGAAAACATTGGAGG-3'
PePCF10-EAR R	5'-CCCGGGGGAGTCGCTGGTGCTCATCGTATC-3'
PePCF10-SRDXG in R	5'-TAGATCCAGATCGGAGTCGCTGGT-3'
PeCIN8-EAR F	5'-CCCGGGCATGAAATCCTCCGGCGAGATCTTG-3'
PeCIN8-EAR R	5'-CCCGGGGTGATACGAACTAGAGGAACGAGAAGACAG-3'
PeCIN8-SRDXG in R	5'-GTTCTAGATCCAGATCGTGATACGAACTAGAG-3'
**Used for Y2H**	
PePCF10-Y2H F	5'-CGCTGCAGATGGAAGGAGAAAACATTGGAGG-3'
PePCF10-Y2H R	5'-CGCTGCAGCTAGGAGTCGCTGGTGCTCATCG-3'
PeCIN8-Y2H F	5'-CCCGGGTATGAAATCCTCCGGCGAGATCTTG-3'
PeCIN8-Y2H R	5'-CCCGGGCCTAGTGATACGAACTAGAGGAACGAGAAGACA-3'

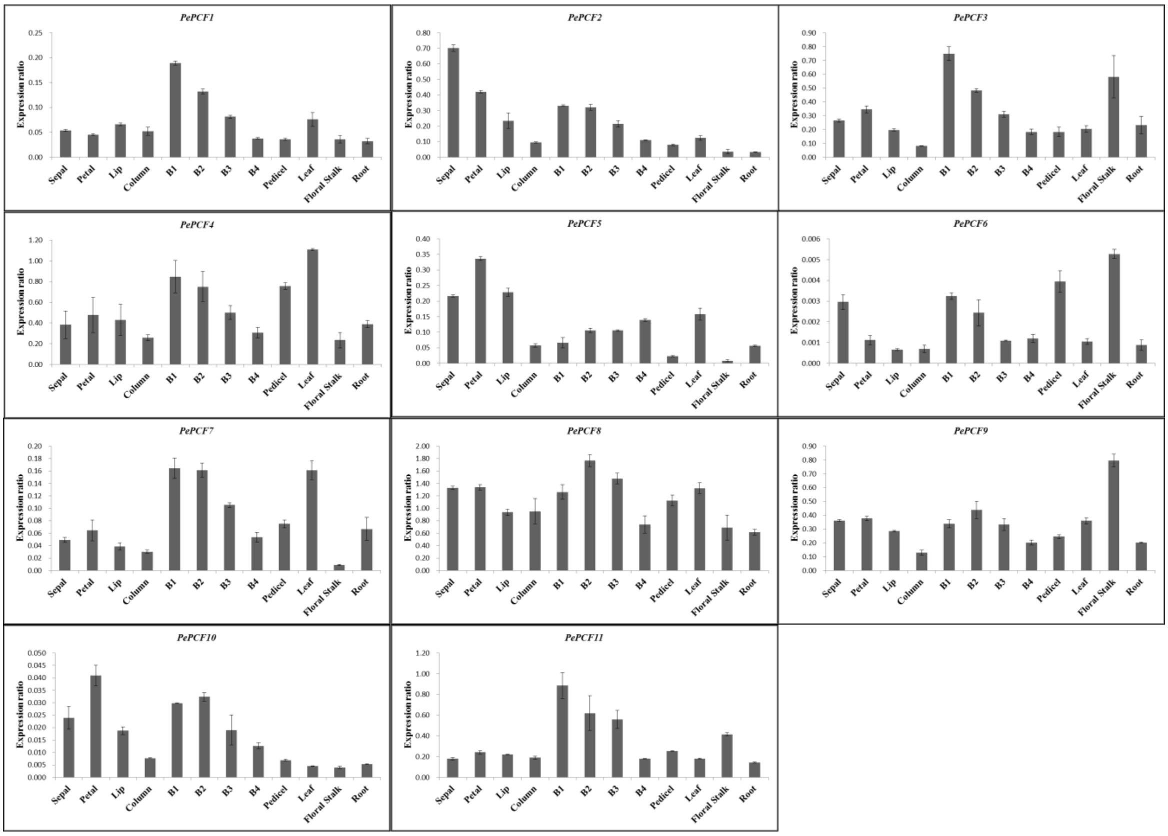
PePCF1
PePCF2
PePCF3
PePCF4
PePCF5
PePCF6
PePCF7
PePCF8
PePCF9
PePCF10
PePCF11
Expression ratio
Sepal
Petal
Lip
Column
B1
B2
B3
B4
Pedicel
Leaf
Floral Stalk
Root

Figure S1

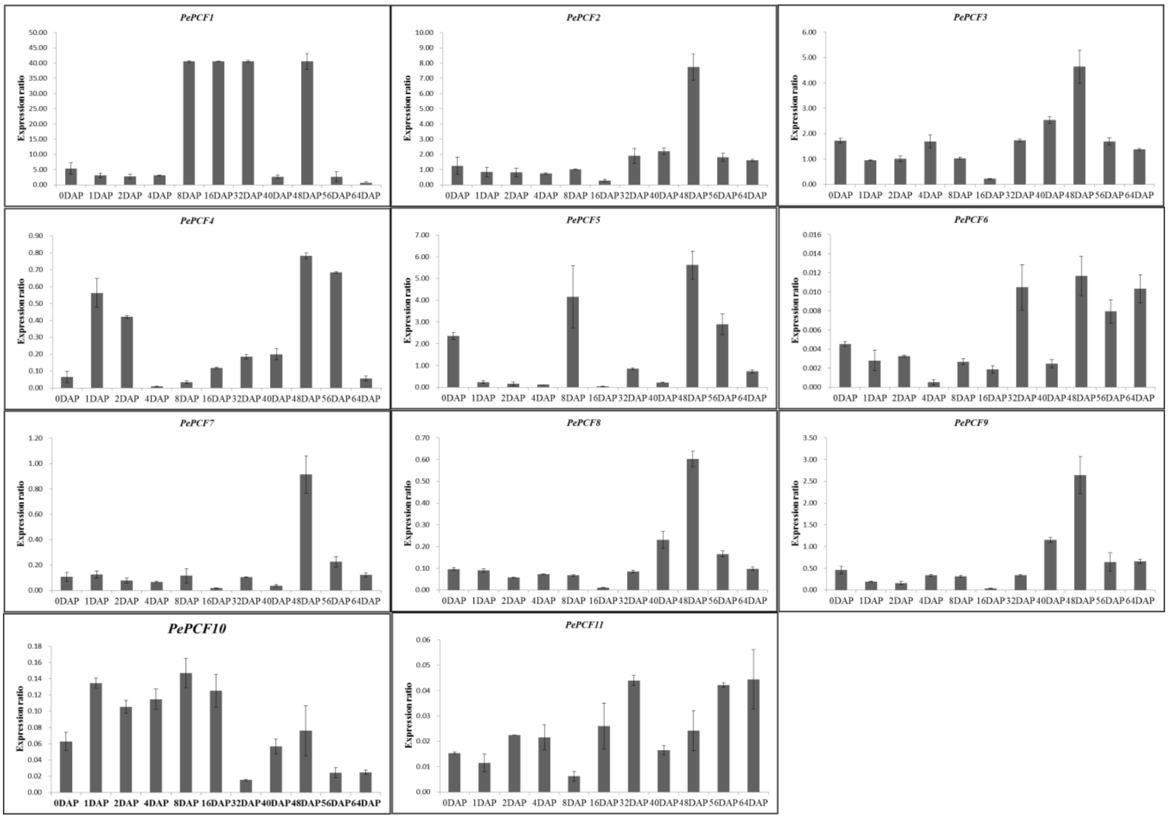
PePCF1
PePCF2
PePCF3
PePCF4
PePCF5
PePCF6
PePCF7
PePCF8
PePCF9
PePCF10
PePCF11
Expression ratio
0DAP 1DAP 2DAP 4DAP 8DAP 16DAP 32DAP 40DAP 48DAP 56DAP 64DAP

Figure S2

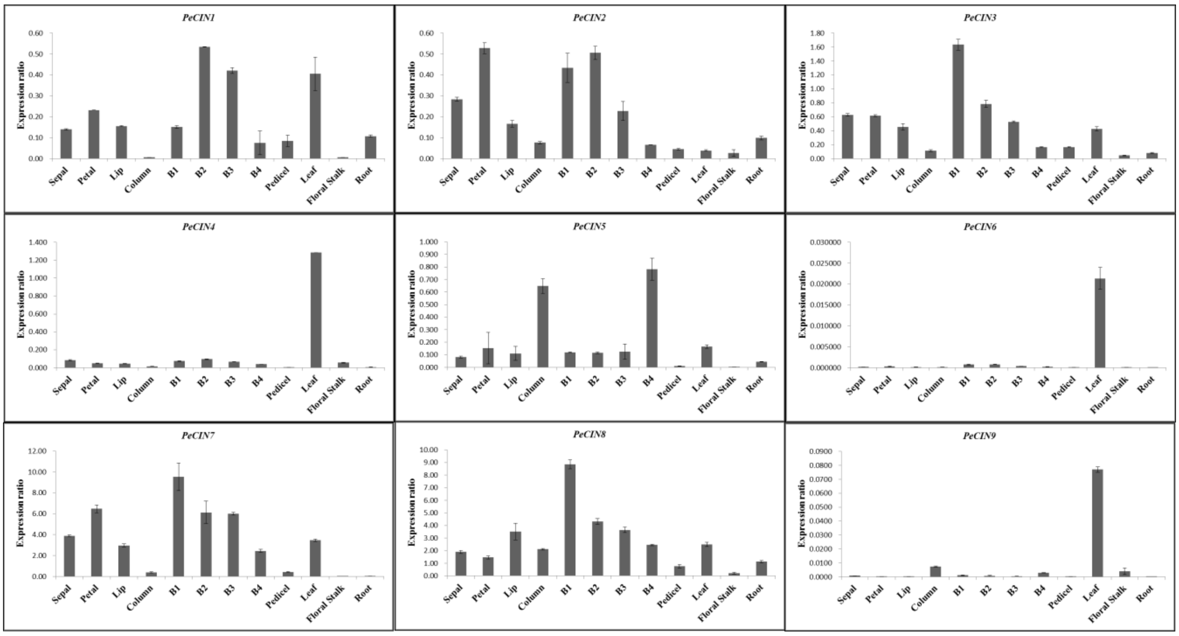
PeCIN1
PeCIN2
PeCIN3
PeCIN4
PeCIN5
PeCIN6
PeCIN7
PeCIN8
PeCIN9
Expression ratio
Sepal
Petal
Lip
Column
B1
B2
B3
B4
Pedicel
Leaf
Floral Stalk
Root

Figure S3

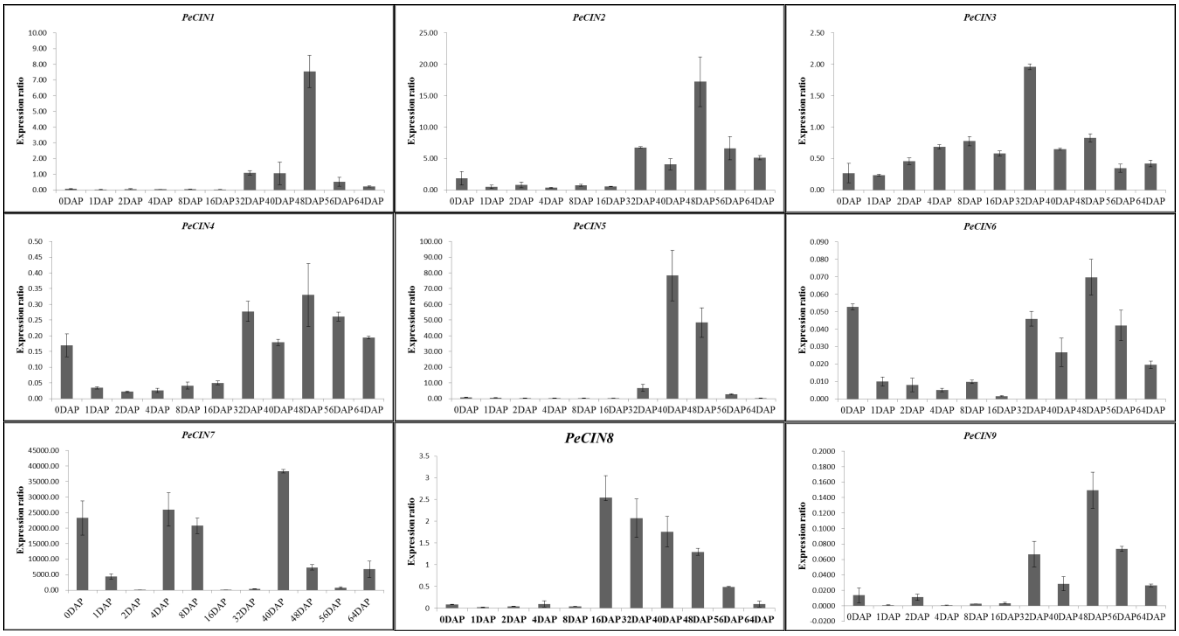
PeCIN1
PeCIN2
PeCIN3
PeCIN4
PeCIN5
PeCIN6
PeCIN7
PeCIN8
PeCIN9
Expression ratio
0DAP
1DAP
2DAP
4DAP
8DAP
16DAP
32DAP
40DAP
48DAP
56DAP
64DAP

Figure S4

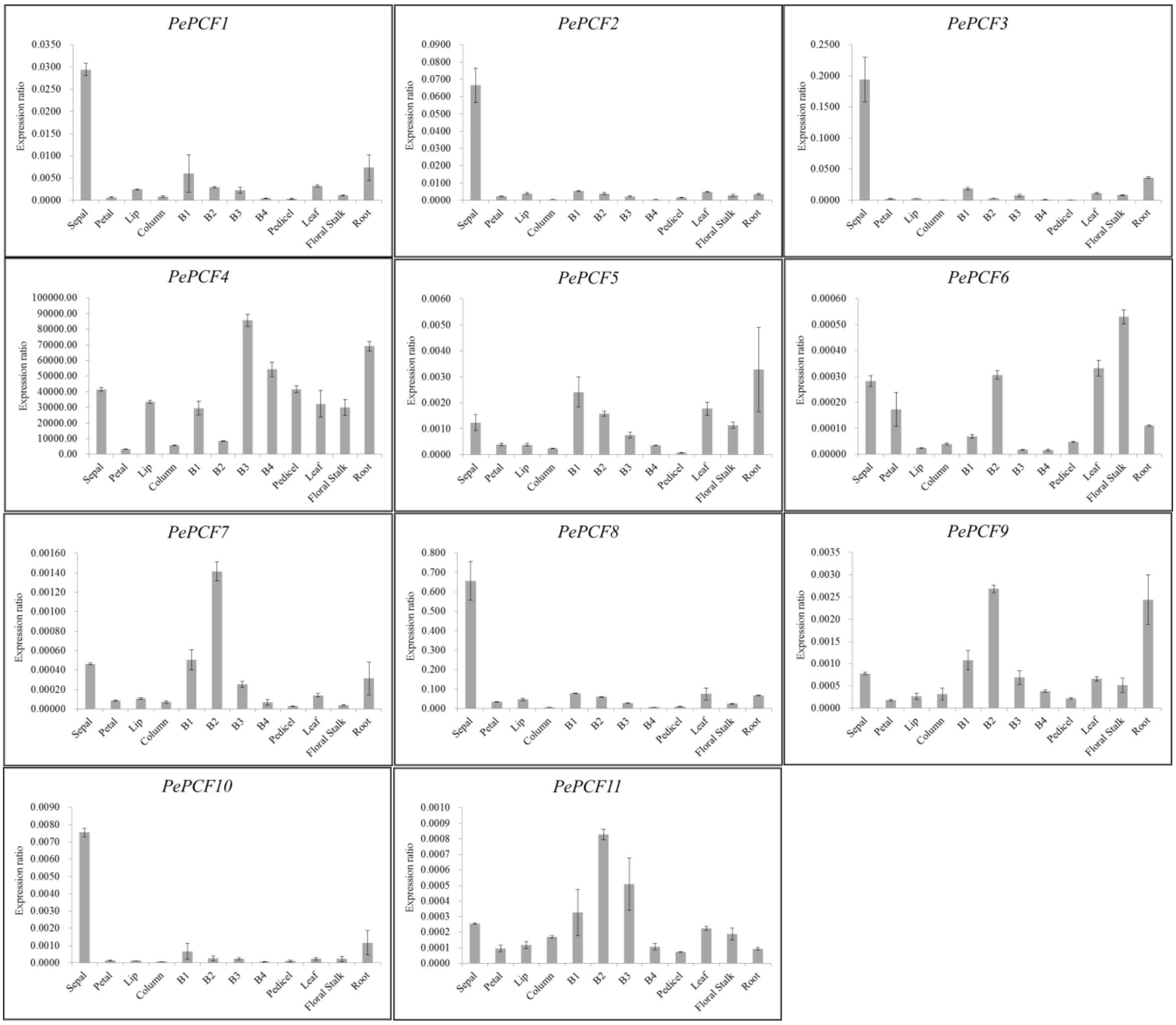

PePCF1
Expression ratio
0.0350
0.0300
0.0250
0.0200
0.0150
0.0100
0.0050
0.0000
Sepal
Petal
Lip
Column
B1
B2
B3
B4
Pedicel
Leaf
Floral Stalk
Root
PePCF2
Expression ratio
0.0900
0.0800
0.0700
0.0600
0.0500
0.0400
0.0300
0.0200
0.0100
0.0000
PePCF3
Expression ratio
0.2500
0.2000
0.1500
0.1000
0.0500
0.0000
PePCF4
Expression ratio
100000.00
90000.00
80000.00
70000.00
60000.00
50000.00
40000.00
30000.00
20000.00
10000.00
0.00
PePCF5
Expression ratio
0.0060
0.0050
0.0040
0.0030
0.0020
0.0010
0.0000
PePCF6
Expression ratio
0.00060
0.00050
0.00040
0.00030
0.00020
0.00010
0.00000
PePCF7
Expression ratio
0.00160
0.00140
0.00120
0.00100
0.00080
0.00060
0.00040
0.00020
0.00000
PePCF8
Expression ratio
0.800
0.700
0.600
0.500
0.400
0.300
0.200
0.100
0.000
PePCF9
Expression ratio
0.0035
0.0030
0.0025
0.0020
0.0015
0.0010
0.0005
0.0000
PePCF10
Expression ratio
0.0090
0.0080
0.0070
0.0060
0.0050
0.0040
0.0030
0.0020
0.0010
0.0000
PePCF11
Expression ratio
0.0010
0.0009
0.0008
0.0007
0.0006
0.0005
0.0004
0.0003
0.0002
0.0001
0.0000

Figure S5

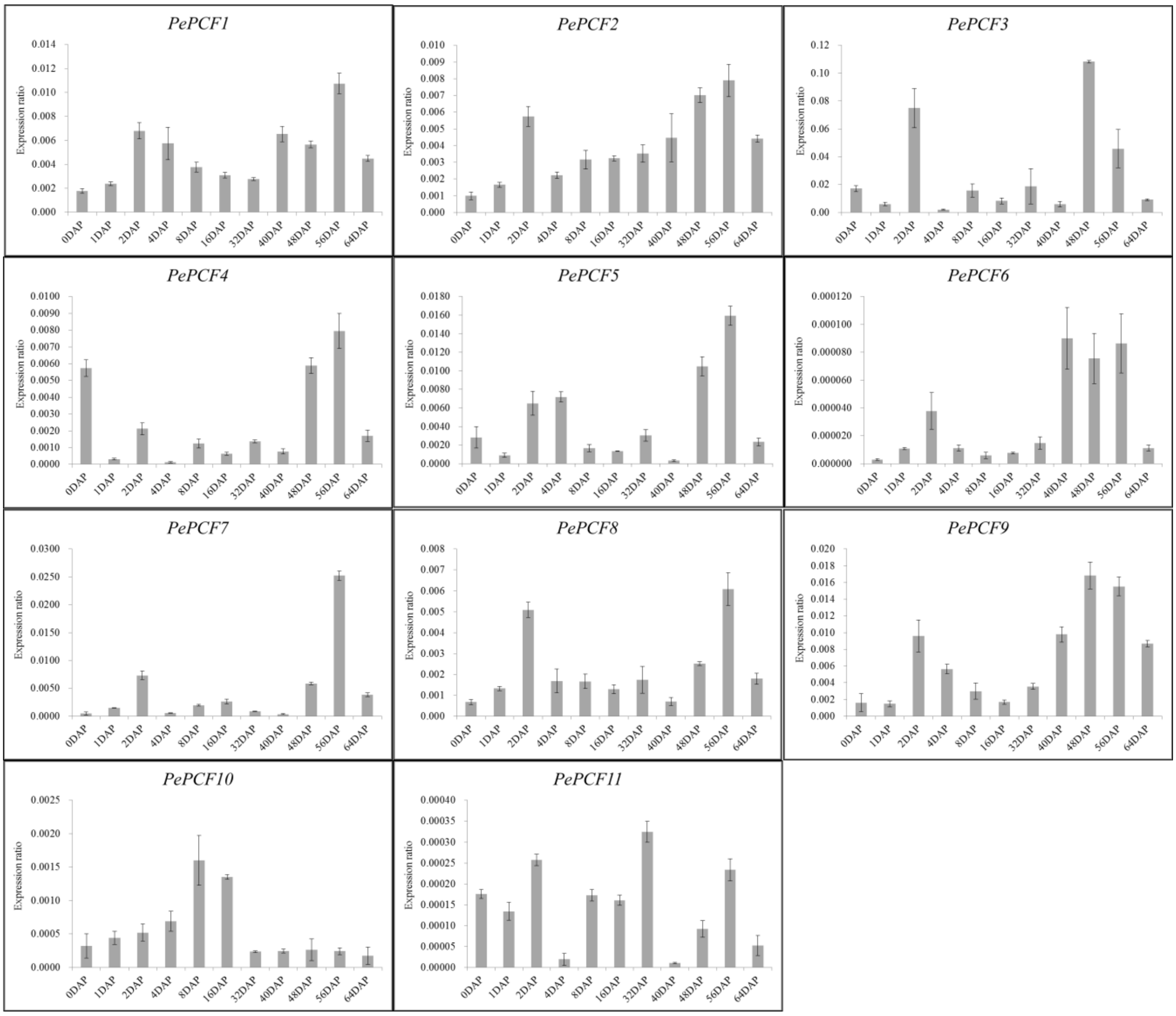
PePCF1
PePCF2
PePCF3
PePCF4
PePCF5
PePCF6
PePCF7
PePCF8
PePCF9
PePCF10
PePCF11
Expression ratio
0DAP 1DAP 2DAP 4DAP 8DAP 16DAP 32DAP 40DAP 48DAP 56DAP 64DAP

Figure S6

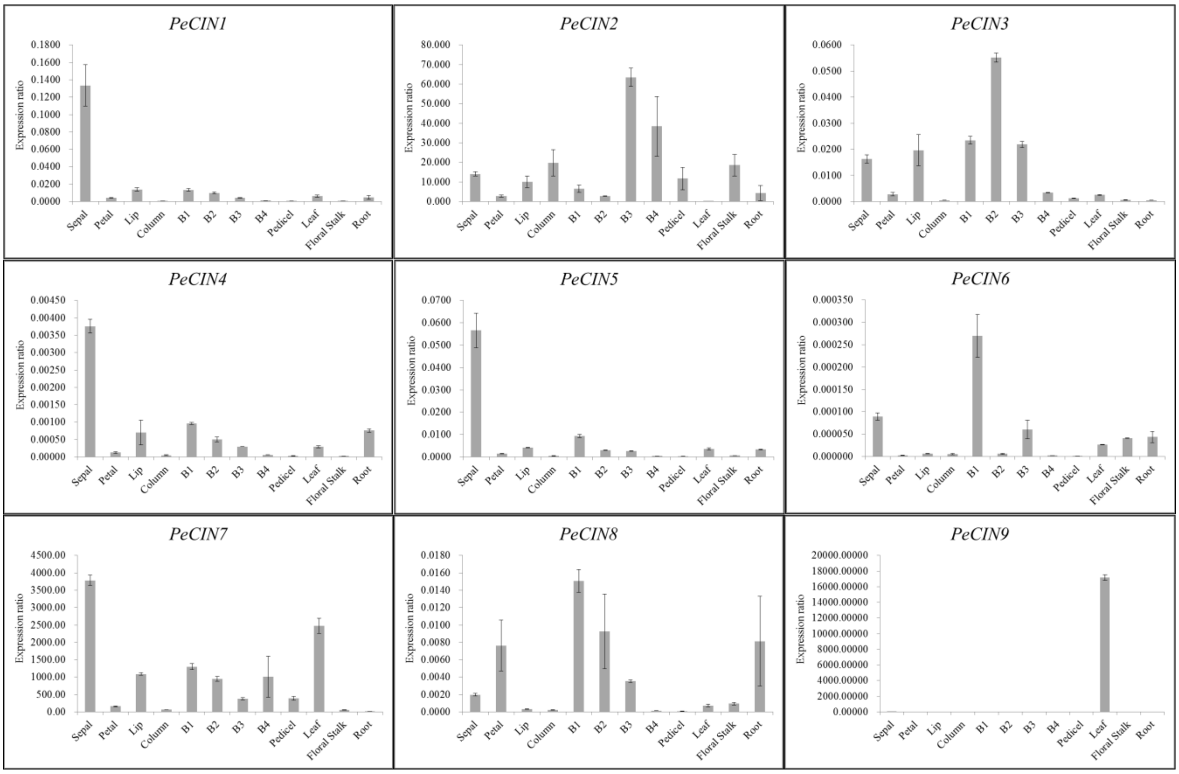
PeCIN1
PeCIN2
PeCIN3
PeCIN4
PeCIN5
PeCIN6
PeCIN7
PeCIN8
PeCIN9
Expression ratio
Sepal
Petal
Lip
Column
B1
B2
B3
B4
Pedicel
Leaf
Floral Stalk
Root

Figure S7

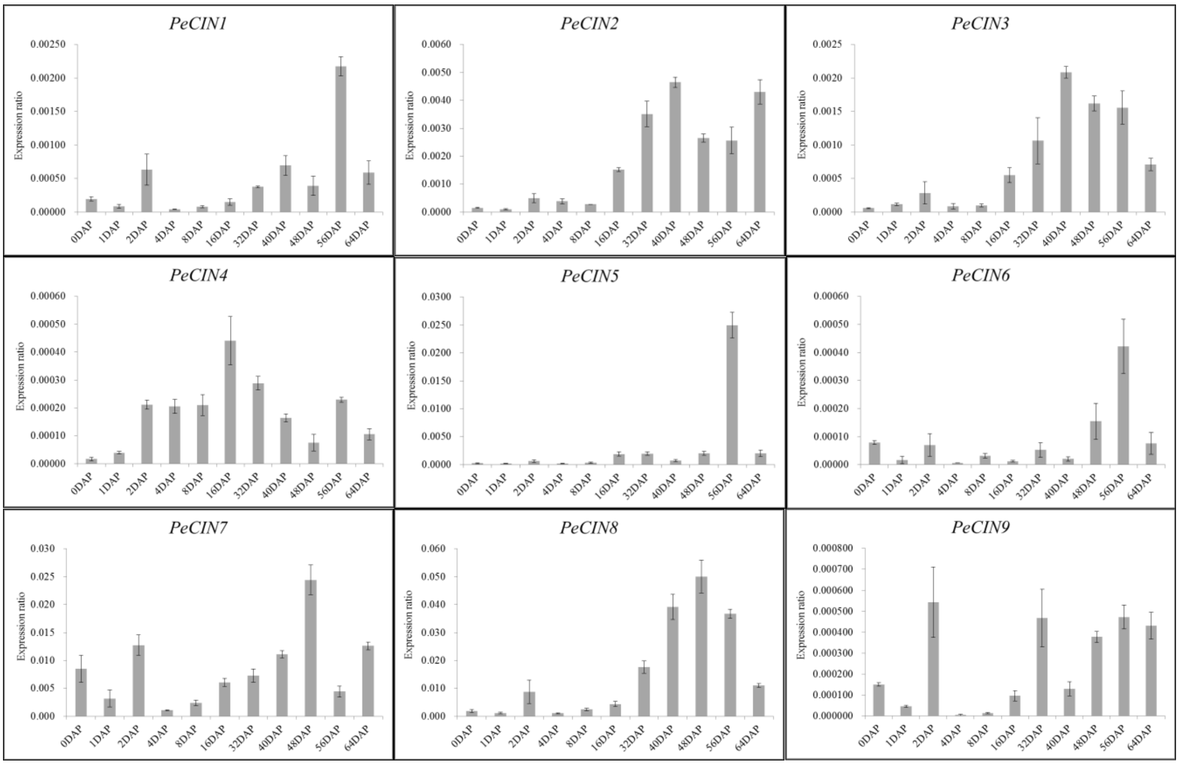
PeCIN1
PeCIN2
PeCIN3
PeCIN4
PeCIN5
PeCIN6
PeCIN7
PeCIN8
PeCIN9
Expression ratio

Figure S8

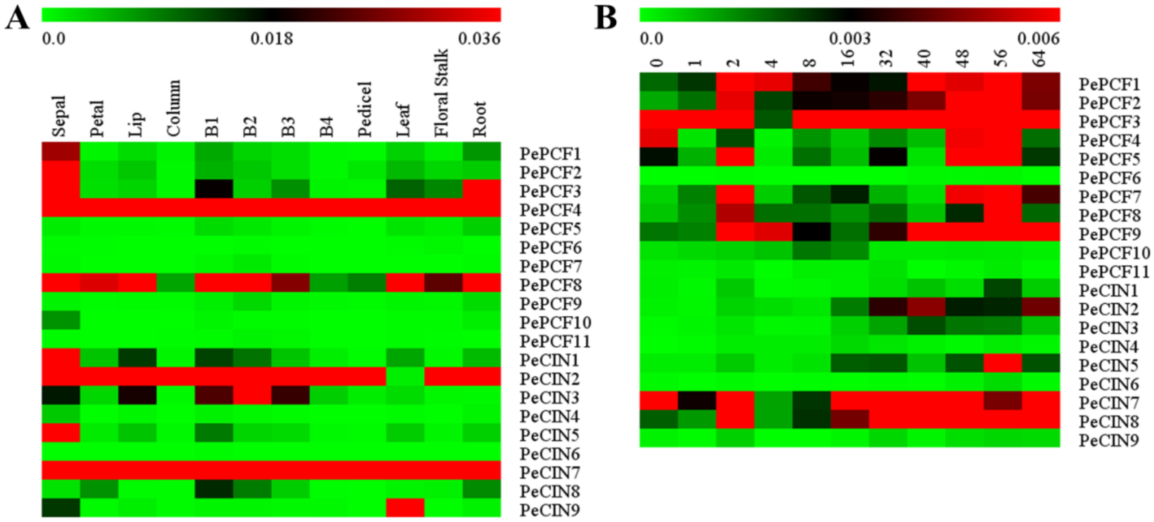
A
0.0
0.018
0.036
Sepal
Petal
Lip
Column
B1
B2
B3
B4
Pedicel
Leaf
Floral Stalk
Root
PePCF1
PePCF2
PePCF3
PePCF4
PePCF5
PePCF6
PePCF7
PePCF8
PePCF9
PePCF10
PePCF11
PeCIN1
PeCIN2
PeCIN3
PeCIN4
PeCIN5
PeCIN6
PeCIN7
PeCIN8
PeCIN9
B
0.0
0.003
0.006
0
1
2
4
8
16
32
40
48
56
64
PePCF1
PePCF2
PePCF3
PePCF4
PePCF5
PePCF6
PePCF7
PePCF8
PePCF9
PePCF10
PePCF11
PeCIN1
PeCIN2
PeCIN3
PeCIN4
PeCIN5
PeCIN6
PeCIN7
PeCIN8
PeCIN9

Figure S9

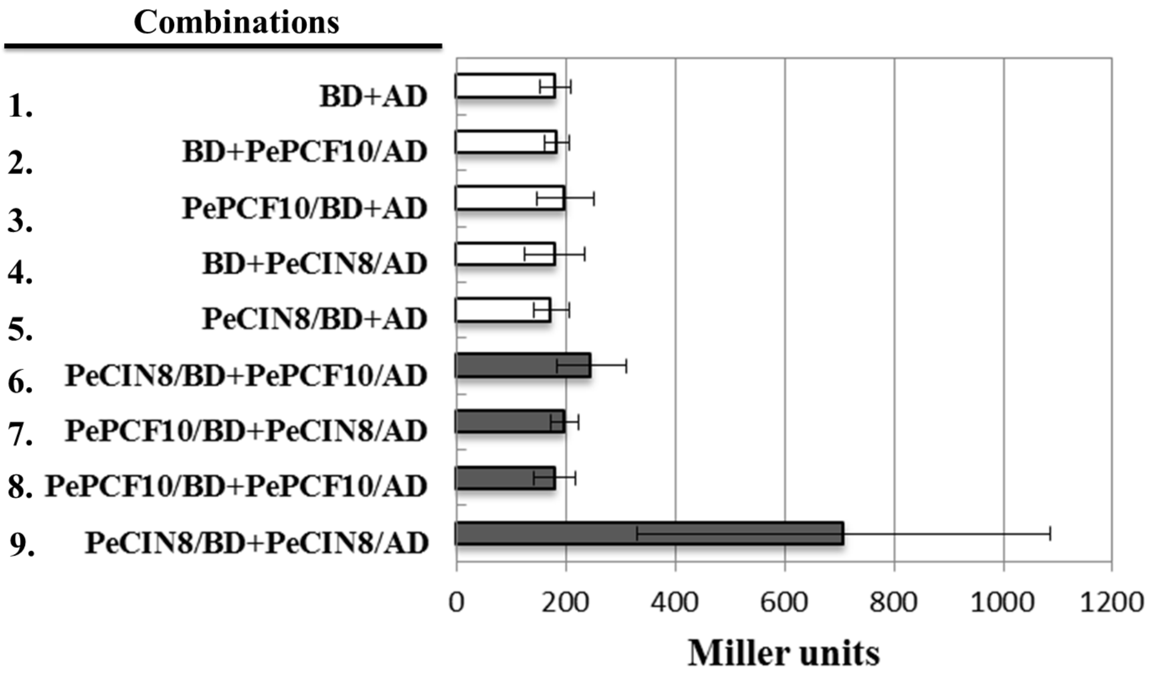
Combinations
1. BD+AD
2. BD+PePCF10/AD
3. PePCF10/BD+AD
4. BD+PeCIN8/AD
5. PeCIN8/BD+AD
6. PeCIN8/BD+PePCF10/AD
7. PePCF10/BD+PeCIN8/AD
8. PePCF10/BD+PePCF10/AD
9. PeCIN8/BD+PeCIN8/AD
0
200
400
600
800
1000
1200
Miller units

Figure S10

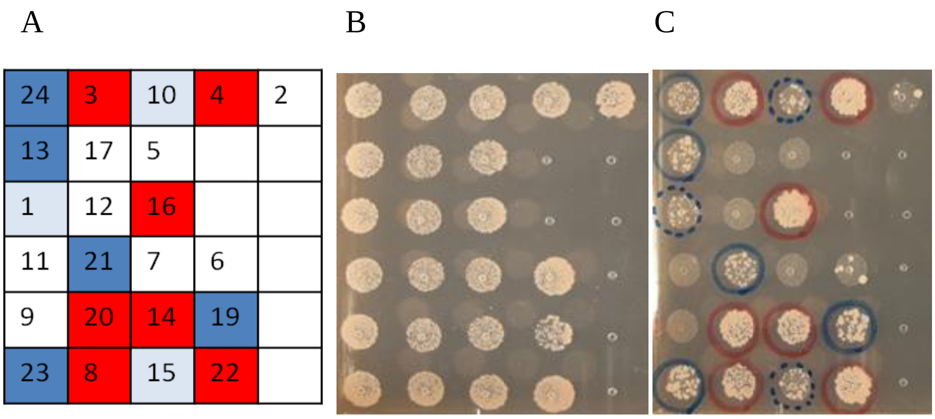

Figure S11
